# Higher premorbid serum testosterone predicts COVID-19-related mortality risk in men

**DOI:** 10.1530/EJE-22-0104

**Published:** 2022-05-10

**Authors:** Bu B Yeap, Ross J Marriott, Laurens Manning, Girish Dwivedi, Graeme J Hankey, Frederick C W Wu, Jeremy K Nicholson, Kevin Murray

**Affiliations:** 1 Medical School, University of Western Australia, Perth, Australia; 2 Department of Endocrinology and Diabetes, Fiona Stanley Hospital, Perth, Australia; 3 School of Population and Global Health, University of Western Australia, Perth, Australia; 4 Department of Infectious Diseases, Fiona Stanley Hospital, Perth, Australia; 5 Harry Perkins Institute of Medical Research, Perth, Australia; 6 Division of Endocrinology, Diabetes & Gastroenterology, School of Medical Sciences, University of Manchester, Manchester, UK; 7 Australian National Phenome Centre, Health Futures Institute, Murdoch University, Perth, Australia; 8 Institute of Global Health Innovation, Imperial College London, London, UK

## Abstract

**Objective:**

Men are at greater risk from COVID-19 than women. Older, overweight men, and those with type 2 diabetes, have lower testosterone concentrations and poorer COVID-19-related outcomes. We analysed the associations of premorbid serum testosterone concentrations, not confounded by the effects of acute SARS-CoV-2 infection, with COVID-19-related mortality risk in men.

**Design:**

This study is a United Kingdom Biobank prospective cohort study of community-dwelling men aged 40–69 years.

**Methods:**

Serum total testosterone and sex hormone-binding globulin (SHBG) were measured at baseline (2006–2010). Free testosterone values were calculated (cFT). the incidence of SARS-CoV-2 infections and deaths related to COVID-19 were ascertained from 16 March 2020 to 31 January 2021 and modelled using time-stratified Cox regression.

**Results:**

In 159 964 men, there were 5558 SARS-CoV-2 infections and 438 COVID-19 deaths. Younger age, higher BMI, non-White ethnicity, lower educational attainment, and socioeconomic deprivation were associated with incidence of SARS-CoV-2 infections but total testosterone, SHBG, and cFT were not. Adjusting for potential confounders, higher total testosterone was associated with COVID-19-related mortality risk (overall trend *P* = 0.008; hazard ratios (95% CIs) quintile 1, Q1 vs Q5 (reference), 0.84 (0.65–1.12) Q2:Q5, 0.82 (0.63–1.10); Q3:Q5, 0.80 (0.66–1.00); Q4:Q5, 0.82 (0.75–0.93)). Higher SHBG was also associated with COVID-19 mortality risk (*P* = 0.008), but cFT was not (*P* = 0.248).

**Conclusions:**

Middle-aged to older men with the highest premorbid serum total testosterone and SHBG concentrations are at greater risk of COVID-19-related mortality. Men could be advised that having relatively high serum testosterone concentrations does not protect against future COVID-19-related mortality. Further investigation of causality and potential underlying mechanisms is warranted.

## Introduction

Infections with severe acute respiratory syndrome coronavirus 2 (SARS-CoV-2) have caused a global pandemic of coronavirus disease 2019 (COVID-19). Vaccinations are effective at reducing risk of infections, and also the severity of breakthrough COVID-19, but coverage needs to be optimised and new variants pose ongoing challenges (1). Men have approximately two-fold higher rates of COVID-19-related mortality than women after adjusting for other risk factors, prompting interest in the potential role of sex hormones in susceptibility to SARS-CoV-2 infection and pathogenesis of COVID-19 (2, 3, 4, 5, 6).

In addition to male sex, older age, obesity, and diabetes are associated with increased risk of intensive care admission and death from COVID-19 (5, 6, 7, 8, 9). As men age, testosterone concentrations decline (10). There is a bidirectional association such that men with higher testosterone concentrations are less likely to be obese or to have type 2 diabetes, and vice versa (11, 12, 13). Respiratory failure is a major contributor to COVID-19 mortality, and higher testosterone concentrations are associated with better indices of lung function in men (14, 15). Conversely, testosterone may have negative effects on the immune system and influences the expression of transmembrane serine protease 2 (TMPRSS2) which facilitates SARS-CoV-2 entry via angiotensin-converting enzyme 2 (ACE2) expressed on cell surfaces (16, 17).

In middle to older-aged men diagnosed or hospitalised with COVID-19, testosterone concentrations are reduced, in proportion to increasing disease severity, expectedly reflecting inhibition of hypothalamic–pituitary–testicular (HPT) axis function during acute illness (18, 19, 20). Knowing whether premorbid testosterone concentration, measured long before exposure to SARS-CoV-2 and therefore not confounded by the acute effects of illness on the HPT axis, is an independent predictor for COVID-19-related mortality risk in men could provide important insights into risk stratification and novel therapeutic interventions.

Circulating testosterone is primarily bound to sex hormone-binding globulin (SHBG), and partly to albumin. Total testosterone and SHBG concentrations are correlated but may have divergent associations with specific outcomes (21). Unbound or free testosterone can be calculated from total testosterone and SHBG, but its utility for predicting health outcomes remains uncertain (22). Given the influence of male sex on COVID-19 mortality risk, an influence of sex hormones on either the incidence or severity of SARS-CoV-2 infection is plausible. However, data on premorbid testosterone concentrations, not confounded by the effects of acute COVID-19 illness, and risk of these outcomes are lacking. We analysed the associations of total testosterone, SHBG, and calculated free testosterone (cFT), assessed in UK Biobank men some years prior to the pandemic, with incidence of SARS-CoV-2 infection and death related to COVID-19. We tested the hypotheses that premorbid total testosterone concentrations in men are associated with incidence rates of SARS-CoV-2 infection and risk of death from COVID-19. We further explored the associations of SHBG and cFT with these outcomes.

## Methods

### The United Kingdom Biobank

UK Biobank recruited community-dwelling adults aged 40–69 years across 22 assessment centres in England, Scotland, and Wales, during 2006–2010 (23). The study was approved by the North West Multi-Centre Research Ethics Committee (reference 06/MRE08/65), and all participants provided informed consent.

### Variables of interest

#### Exposures

Blood samples were collected at the initial baseline visit (March 2006–October 2010). Blood collection occurred throughout the course of the day, following which the specimen collection tubes were swiped and collection time was registered. Serum samples were prepared and stored at −80°C until assayed in the UK Biobank central laboratory (24, 25). Serum total testosterone was assayed using a competitive binding chemiluminescent immunoassay (DXI 800, Beckman Coulter, High Wycombe, UK: analytical range, 0.35–55.5 nmol/L (10–1599 ng/dL); coefficients of variation, 8.3, 3.7, and 4.2% for low, medium, and high ranges (1.0–2.2, 13.4–22.8, and 29.3–49.4 nmol/L or 29–63, 386–657, and 844–1424 ng/dL)). Serum SHBG was assayed using a two-step sandwich chemiluminescent immunoassay (DXI 800, Beckman Coulter: analytical range, 0.33–242 nmol/L; coefficients of variation, 5.7, 5.3, and 5.2% for low, medium, and high ranges (15.0–27.7, 31.9–55.5, and 56.3–87.8 nmol/L)). Free testosterone (cFT) was calculated using the Vermeulen method from total testosterone and SHBG, with fixed albumin concentration (42 g/L) (22).

#### Study outcomes

Associations with the incidence of reported SARS-CoV-2 infections were investigated in exploratory analyses. Deaths related to COVID-19 were investigated as a primary outcome. Follow-up was commenced on 16 March 2020, the date from which testing data were routinely provided to UK Biobank (Supplementary Methods, see section on supplementary materials given at the end of this article). End of follow-up was selected as 31 January 2021, before vaccinations were widely available to the UK population (Supplementary Table 1). Sensitivity analyses investigated alternative end-of-follow-up dates: 8 December 2021 when vaccinations commenced in the UK, and 28 February 2021, towards the tail of the second wave, and the latest date to which death and cause of death data were complete.

Positive PCR test results for SARS-CoV-2 were captured by Public Health England’s Second Generation Surveillance System, Public Health Scotland, and, for Wales, by the Secure Anonymised Information Linkage and provided to UK Biobank (26). Test results were collected by health care providers in hospital, emergency department, and community settings. Incident events of reported SARS-CoV-2 infections were identified as either the first positive PCR test or death from COVID-19 without a prior positive test result during follow-up (26). Monthly incidence rates (per 1000 person-months) were calculated (Supplementary Methods). COVID-19 deaths were identified by the listing of ICD-10 codes U07.1, U07.2 anywhere on the death certificate. Individuals who did not record an incident event were censored at the earliest of their date(s) of non-COVID-19-related death or end of follow-up.

#### Other variables of interest

Social, demographic, and lifestyle variables: educational attainment, alcohol consumption, dietary intake, physical activity, ethnicity, and socioeconomic status (Townsend Index) were categorised, and assessment centres were grouped into geographic regions (Supplementary Methods). Prevalent medical conditions were defined by self-report, International Classification of Diseases (ICD) diagnosis codes (Supplementary Table 2) from previous hospital inpatient admissions, cancer registry data and by physical and biochemical measurements (e.g. for blood pressure and glucose concentrations), and medications usage was recorded and categorised (Supplementary Methods).

### Statistical analysis

The pattern of infections varied across regions during follow-up (Supplementary Methods and Supplementary Fig. 1). Accordingly, all analyses included an interaction term of spatial unit with time. Monthly incidence rates were modelled (Supplementary Methods). The response variable was the count of incident SARS-CoV-2 infections and predictors included the exposure hormone as quintile categories, age category, BMI category, country (England, Scotland, Wales), educational qualifications, ethnicity, Townsend index quintile, month, and number at risk as a logged offset term. Month was modelled using a natural cubic spline with a knot point set on the day preceding the introduction of the ‘Rule of 6’ social distancing measure (14 September 2020), the first of a series of national restrictions introduced to address the second wave of the epidemic. The follow-up periods before and after this date are referred to as ‘Wave 1’ and ‘Wave 2’, respectively. An interaction term of country with month was included. Incidence rate ratios (IRRs) and 95% CIs were calculated from each of the fitted models for categorical predictors of interest, including the exposure variable (hormone quintile), and sensitivity analyses were conducted to evaluate alternative end-of-follow-up dates (Supplementary Methods).

COVID-19 deaths were modelled using time-stratified Cox regression, initially with two time strata set to delineate ‘Wave 1’ from ‘Wave 2’. Each analysis involved fitting two models. A minimally adjusted model (model 1) included the exposure variable, baseline age, UK region, and a region with time stratum interaction (Supplementary Methods). The fully adjusted model (model 2) was adjusted for additional covariates: lifestyle and demographic variables (ethnicity, living with partner, alcohol consumption, diet, physical activity, educational attainment, smoking status, waist circumference, BMI, cholesterol), time of day for blood sampling (decimal hours), blood type, blood cholesterol, prevalent medical conditions (history of angina, atrial fibrillation, cancer, cardiovascular disease, chronic obstructive pulmonary disease (COPD), diabetes, hypertension, liver disease, renal impairment, thyroid disease), and prevalent medication usage (anticonvulsants, lipid, glucocorticoids, opioids). The total number of medications was included as a proxy for comorbidity status. We investigated a third model including terms for both total testosterone and SHBG, but this was not pursued because of a relatively high negative correlation (r = −0.57) between estimated testosterone and SHBG coefficients. Continuous predictors were modelled using restricted cubic splines, and validity of the proportional hazards assumption was assessed using per-variable and global tests (Supplementary Methods).

Statistical significance of associations was evaluated using likelihood ratio tests, comparing the full model to that without the exposure term. Accordingly, *P* values are for the overall trend, with a threshold of *P* < 0.05 regarded as significant. Hazard ratios (HRs) and 95% CIs were calculated from each of the fitted models, relative to the median of the fifth sample quintile as the reference value, and plotted against the exposure variable over a continuous range to show non-linear effects in figures (Supplementary Methods). HRs and 95% CIs associated with the change in hormone concentration from this reference value to the median of each of the other sample quintiles were tabulated. Analyses for total testosterone were replicated for SHBG, and cFT, as additional exposures of interest. Sensitivity analyses (model 2) were conducted to evaluate alternative end-of-follow-up dates (Supplementary Methods).

## Results

### Study cohort

Of 229 106 male UK Biobank participants assessed in 2006–2010, excluding those who died, were lost to follow-up (*n* = 605, 0.26%), 2 with prior infection, and those with diseases or medications affecting testosterone concentrations, left 206 722 men (Supplementary Fig. 2). Further excluding for missing testosterone (*n* = 14 309) or SHBG (*n* = 28 698) or other baseline measurements left 159 964 men for the analysis. Excluded men had a slightly higher proportion of current smokers and men with CVD and diabetes (Supplementary Table 3).

### Baseline characteristics of the study cohort

Totally 24 175 men were tested for and 5 558 were infected with SARS-CoV-2, with 438 deaths from COVID-19. Men diagnosed with SARS-CoV-2 infection were slightly younger, and those who died from COVID-19 were substantially older, than the cohort as a whole (Table 1). Men who died from COVID-19 had higher BMI, were less physically active, more likely to be previous or current smokers, have medical comorbidities such as hypertension, cardiovascular disease, diabetes, cancer, COPD, atrial fibrillation, renal impairment, on lipid medications, and taking ≥5 medications, compared to men infected with SARS-CoV-2 and the cohort as a whole (Table 1 and Supplementary Table 4).

**Table 1:** Baseline characteristics of UK Biobank men, stratified according to those who were tested for infection, or were infected with or died from COVID-19 during the follow-up period and for the cohort as a whole^**^.

Basic Characteristics^*,§^	Participants with this event recorded duing follow-up			All^**^
	Tested	Infected with SARS-CoV-2^§§^	Died from COVID-19	
Sociodemographic and lifestyle				
*n*	24 175	5558	438	159 964
Age (whole years)	59.0 (50.0–64.0)	54.0 (46.0–62.0)	65.0 (61.0–67.0)	57.0 (50.0–63.0)
BMI (kg/m^2^)	27.8 (25.4–30.6)	28.0 (25.7–30.9)	28.8 (26.1–32.5)	27.2 (25.0–29.9)
Waist circumference (cm)	97.0 (90.0–105.0)	97.0 (91.0–105.0)	102.0 (94.0–111.0)	96.0 (89.0–103.0)
Country				
England	86.2 (20 839)	90.9 (5052)	85.4 (374)	88.7 (141 850)
Scotland	8.2 (1981)	4.4 (246)	8.9 (39)	7.0 (11 140)
Wales	5.6 (1355)	4.7 (260)	5.7 (25)	4.4 (6974)
Townsend Index				
Q1	19.7 (4758)	17.1 (951)	17.8 (78)	20.6 (32 949)
Q2	19.8 (4786)	17.7 (983)	15.8 (69)	20.5 (32 717)
Q3	19.6 (4740)	19.3 (1071)	18.3 (80)	20.4 (32 629)
Q4	19.5 (4723)	20.6 (1144)	18.5 (81)	19.7 (31 557)
Q5	21.4 (5168)	25.4 (1409)	29.7 (130)	18.8 ( 30 112)
Ethnicity: not White	5.2 (1267)	8.5 (475)	5.9 (26)	4.8 (7717)
Qualifications: college/university	31.7 (7654)	26.8 (1487)	23.1 (101)	36.1 (57 680)
Partner: true	78.3 (18 923)	78.4 (4356)	68.3 (299)	78.3 (125 271)
Alcohol consumption				
Low	41.0 (9901)	42.7 (2376)	45.2 (198)	40.5 (64 850)
Medium	28.9 (6992)	28.2 (1565)	28.8 (126)	29.6 (47 376)
High	30.1 (7282)	29.1 (1617)	26.0 (114)	29.8 (47 738)
Diet				
High red meat eaters	17.1 (4130)	16.3 (908)	19.4 (85)	16.0 (25 561)
Low red meat eaters	80.0 (19 338)	80.3 (4465)	78.3 (343)	80.5 (128 826)
No red meat	2.9 (707)	3.3 (185)	2.3 (10)	3.5 (5577)
PA				
Insufficient	40.7 (9839)	39.1 (2175)	46.3 (203)	39.9 (63 768)
Sufficient	15.6 (3762)	15.6 (869)	17.1 (75)	15.9 (25 403)
Additional	43.7 (10 574)	45.2 (2514)	36.5 (160)	44.3 (70 793)
Smoking				
Never	46.4 (11 217)	47.7 (2653)	30.4 (133)	50.7 (81 042)
Previous	41.1 (9945)	39.8 (2213)	54.8 (240)	38.0 (60 770)
Current	12.5 (3013)	12.5 (692)	14.8 (65)	11.3 (18 152)
Prevalent health conditions and medication usage				
CVD	7.6 (1827)	6.3 (348)	16.7 (73)	5.3 (8516)
Diabetes	9.7 (2336)	9.9 (551)	21.7 (95)	7.0 (11 208)
Cancer	5.6 (1363)	4.2 (235)	6.6 (29)	4.3 (6866)
Angina	7.0 (1699)	5.7 (315)	13.7 (60)	4.9 (7783)
Atrial fibrillation	2.9 (709)	2.6 (146)	8.7 (38)	2.0 (3175)
Renal impairment	1.0 (240)	0.8 (42)	2.1 (9)	0.6 (916)
Hypertension	65.5 (15 830)	60.2 (3346)	83.6 (366)	61.9 (99 002)
COPD	1.1 (268)	0.9 (50)	3.9 (17)	0.6 (984)
Liver disease	1.7 (416)	1.7 (96)	2.1 (9)	1.2 (1937)
Thyroid disease	2.3 (568)	2.2 (123)	3.2 (14)	2.1 (3296)
Lipid medication use	26.7 (6449)	22.8 (1270)	47.0 (206)	22.4 (35 868)
Glucocorticoid use	8.5 (2045)	7.8 (434)	10.7 (47)	7.0 (11 168)
Opioid use	5.9 (1425)	5.4 (301)	11.2 (49)	4.0 (6405)
Anticonvulsant use	1.9 (451)	1.5 (82)	3.4 (15)	1.3 (2109)
Medication, *n*				
0	27.2 (6586)	32.2 (1790)	13.9 (61)	33.3 (53 267)
1–2	31.7 (7662)	32.1 (1783)	20.3 (89)	33.3 (53 337)
3–4	19.7 (4764)	17.0 (945)	17.6 (77)	18.2 (29 166)
5+	21.4 (5163)	18.7 (1040)	48.2 (211)	15.1 (24 194)
Blood/hormone variables				
Time blood drawn (dec hour)	14.5 (11.8–16.9)	14.6 (11.8–17.1)	14.4 (12.0–16.5)	14.5 (11.8–17.0)
Blood type				
A	43.5 (10 514)	45.0 (2500)	40.4 (177)	43.2 (69 153)
B	3.7 (886)	4.1 (229)	3.9 (17)	3.6 (5730)
AB	9.7 (2339)	10.2 (568)	9.6 (42)	9.5 (15 198)
O	43.2 (10 436)	40.7 (2261)	46.1 (202)	43.7 (69 883)
Cholesterol (mmol/L)	5.4 (4.7–6.2)	5.4 (4.7–6.2)	5.1 (4.2–5.8)	5.5 (4.8–6.2)
Testosterone (nmol/L)	11.5 (9.3–14.0)	11.4 (9.3–14.0)	11.0 (8.9–13.4)	11.6 (9.5–14.1)
Testosterone (ng/dL)	331 (268–403)	329 (268–403)	317 (256–386)	334 (274–406)
SHBG (nmol/L)	36.5 (27.4–47.7)	34.2 (25.6–44.7)	38.2 (29.3–51.0)	36.6 (27.7–47.6)
cFT (pmol/L)	212 (176–254)	220 (182–262)	193 (164–233)	215 (180–257)

^*^Continuous variables (age, BMI, cholesterol, waist circumference, time blood drawn, testosterone, SHBG, cFT) represented as median (interquartile range); other variables as percentages (numbers) per category. ^**^Summary data presented for data after excluding men who died or were lost to follow-up since their baseline visit but before 16 March 2020, with prior orchidectomy, taking androgens, anti-androgen, 5α-reductase, estrogen, anti-estrogen, progesterone medications, infertile men, men with pituitary disease, adrenogenital or testicular disorders, or variables with missing values. ^§^BMI(kg/m^2^); Education, Educational attainment; PA, level of physical activity categories (min/week; see Supplementary Methods); Alcohol, level of alcohol consumption (standard units of alcohol consumed/week; see Supplementary Methods); Smoking, smoking status; SHBG, sex hormone binding globulin; cFT, free testosterone calculated using the Vermeulen formula. ^§§^Incident infections identified for participants with a positive test result or who died from COVID-19 during the follow-up period from 16 March 2020 to 31 January 2021.

### Associations of testosterone and SHBG with incidence of SARS-CoV-2 infection

**Figure 1:**
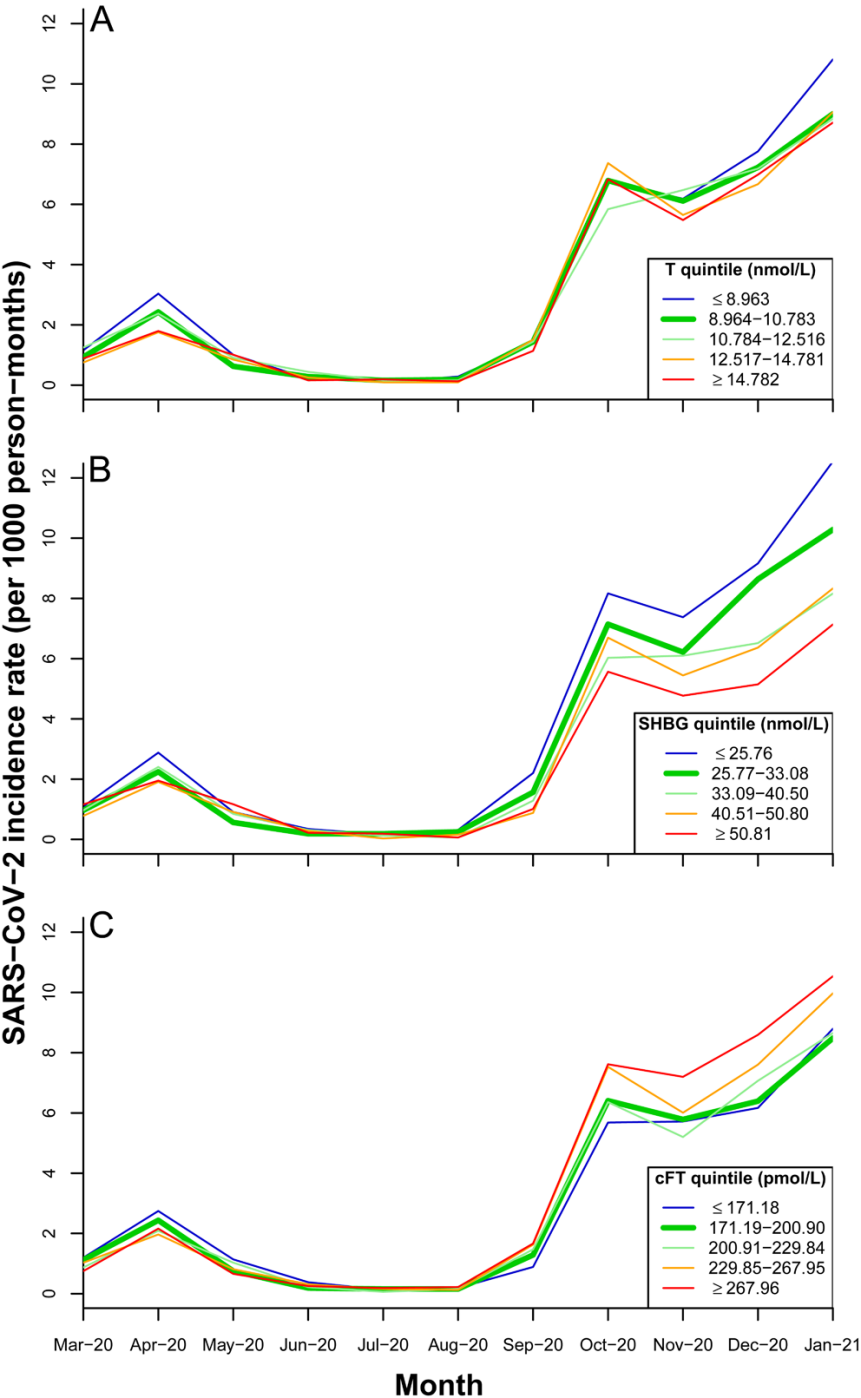
Monthly incidence rate of reported SARS-CoV-2 infections in UK Biobank men grouped by their baseline (2006–2010) serum concentrations of (A) total testosterone, (B) SHBG, and (C) calculated free testosterone (cFT). To convert total testosterone concentrations from nmol/L to mg/dL, divide by 0.0347. A full colour version of this figure is available at https://doi.org/10.1530/EJE-22-0104.

Monthly incidence rates of reported SARS-CoV-2 infections are shown, stratified according to quintiles of each exposure variable (Fig. 1). Infections peaked in April 2020, with a higher second peak in October 2020, then increased steadily from November 2020 onwards. Men with serum total testosterone concentrations in the lowest quintile had marginally higher incidence rates in some months (April 2020, December–January 2021) compared with other men (Fig. 1A). From the second peak in October 2020 onwards, men with lower SHBG concentrations, and men with cFT in the highest quintile, had higher incidence rates (Fig. 1B and C).

In exploratory analyses, there were no associations of total testosterone, SHBG, or cFT with incidence of SARS-CoV-2 infection (Table 2). In each of the models with the different hormone predictors, incidence rate ratios (IRRs) were lower for men who were older and had a higher level of educational attainment and were higher for men with higher BMI, non-White ethnicity, or higher indices of socioeconomic deprivation at baseline (Table 2). Sensitivity analyses excluding men with diabetes or using earlier or later end-of-follow-up dates made no substantive difference to the results (Supplementary Tables 5, 6 and 7).

**Table 2:** Incidence rate ratios (IRRs) and IRR 95% CIs of SARS-CoV-2 infections in UK Biobank men during follow-up (16 March 2020 to 31 January 2021). Estimates presented for model predictors of interest, including for quintile categories of the baseline hormone concentration (testosterone, SHBG, cFT), from Poisson regression.

Predictor	Hormone term modelled as quintile categories^*^		
	Testosterone	SHBG	cFT
Hormone			
Quintile 5 (ref)	1	1	1
Quintile 4	1.01 (0.93–1.11)	1.07 (0.97–1.17)	1.04 (0.96–1.13)
Quintile 3	0.99 (0.90–1.08)	1.02 (0.93–1.12)	0.97 (0.89–1.06)
Quintile 2	0.98 (0.90–1.08)	1.09 (0.99–1.19)	1.01 (0.92–1.10)
Quintile 1	1.04 (0.95–1.14)	1.08 (0.98–1.19)	1.02 (0.93–1.12)
Age			
≤50 (ref)	1	1	1
51–60	0.62 (0.58–0.67)	0.64 (0.60–0.69)	0.62 (0.58–0.66)
>60	0.56 (0.52–0.60)	0.58 (0.54–0.63)	0.56 (0.52–0.60)
BMI			
<25 (ref)	1	1	1
25–<30	1.29 (1.19–1.39)	1.28 (1.18–1.38)	1.28 (1.19–1.38)
≥30	1.63 (1.50–1.77)	1.60 (1.47–1.74)	1.62 (1.49–1.75)
Ethnicity: not White	1.64 (1.48–1.82)	1.64 (1.48–1.82)	1.66 (1.50–1.84)
Qualifications: college/university	0.68 (0.64–0.72)	0.67 (0.63–0.72)	0.67 (0.63–0.72)
Townsend Index			
Quintile 1 (ref)	1	1	1
Quintile 2	1.00 (0.91–1.09)	1.00 (0.91–1.10)	1.00 (0.91–1.09)
Quintile 3	1.07 (0.98–1.17)	1.07 (0.98–1.18)	1.06 (0.97–1.16)
Quintile 4	1.16 (1.06–1.27)	1.13 (1.03–1.23)	1.15 (1.05–1.26)
Quintile 5	1.37 (1.26–1.50)	1.37 (1.25–1.49)	1.37 (1.25–1.49)

^*^Quintile boundaries: testosterone: (nmol/L) Q1/2 9.0, Q2/3 10.8, Q3/4 12.5, and Q4/5 14.8 or (ng/dL) Q1/2 259, Q2/3 311, Q3/4 360, and Q4/5 427; SHBG: (nmol/L) Q1/2 25.8, Q2/3 33.1, Q3/4 40.5, and Q4/5 50.8; cFT: (pmol/L) Q1/2 171, Q2/3 201, Q3/4 230, and Q4/5 268.

### Associations of testosterone and SHBG with COVID-19 deaths

**Figure 2:**
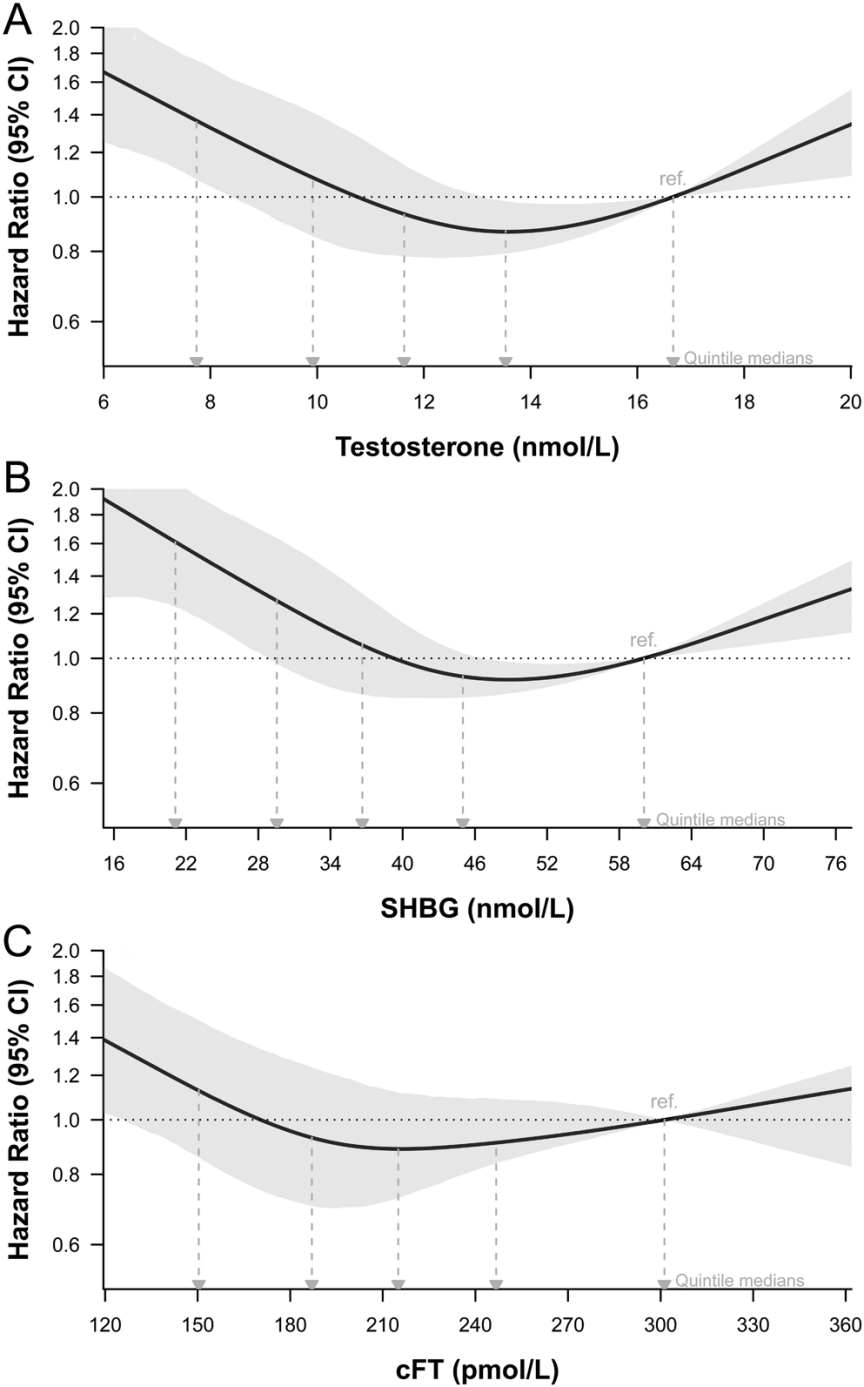
Risk of death from COVID-19, according to baseline serum concentrations of (A) total testosterone, (B) SHBG, and (C) calculated free testosterone (cFT). Model 1: minimally adjusted model. Shaded areas are the 95% CIs. Horizontal plot axes are truncated to exclude values lower or higher than the 2.5th and 97.5th percentiles. The location of hazard ratios for medians of quintiles for each exposure variable is shown as they relate to results in Table 3. To convert total testosterone concentrations from nmol/L to mg/dL, divide by 0.0347.

In the minimally adjusted model (Model 1) which included exposure variable, age, region, and interaction of region with time, total testosterone, SHBG, and cFT were associated with risk of dying from COVID-19 (Table 3, Model 1: *P* values for overall trends <0.05). U-shaped associations of total testosterone and SHBG concentrations with risk of death from COVID-19 were apparent (Fig. 2A and B). A weaker association was seen for cFT (Fig. 2C).

**Table 3:** Hazard ratios estimating the relative risk of death from COVID-19 associated with baseline hormone concentration.^§^

Model	Q1 (lowest)	Q2	Q3	Q4	Q5 (highest)	*P*-value (term)
Total testosterone (nmol/L)						
Events per quintile	112	94	86	68	78	
*n* per quintile	31 992	32 169	32 081	31 933	31 789	
Model 1^#^	1.37 (1.07–1.75)	1.08 (0.85–1.41)	0.93 (0.78–1.14)	0.87 (0.79–0.98)	ref.	<0.001
Model 2^##^	0.84 (0.65–1.12)	0.82 (0.63–1.10)	0.80 (0.66–1.00)	0.82 (0.75–0.93)	ref.	0.008
SHBG (nmol/L)						
Events per quintile	76	79	87	86	110	
*n* per quintile	31 980	32 121	32 054	31 982	31 827	
Model 1^#^	1.61 (1.23–2.07)	1.27 (0.98–1.64)	1.06 (0.86–1.30)	0.93 (0.85–1.02)	ref.	<0.001
Model 2^##^	1.01 (0.77–1.34)	0.94 (0.72–1.24)	0.89 (0.71–1.11)	0.87 (0.79–0.97)	ref.	0.008
cFT (pmol/L)						
Events per quintile	137	110	76	66	49	
*n* per quintile	31 738	32 051	32 126	32 073	31 976	
Model 1^#^	1.13 (0.86–1.5)	0.93 (0.7–1.24)	0.89 (0.73–1.12)	0.91 (0.84–1.09)	ref.	0.004
Model 2^##^	0.86 (0.65–1.17)	0.81 (0.61–1.10)	0.83 (0.68–1.07)	0.89 (0.82–1.06)	ref.	0.248

^§^Hazard ratios calculated for the medians of testosterone within each sample quintile (Q1–Q5), relative to the median for Q5. Quintile boundaries: testosterone: (nmol/L) Q1/2 9.0, Q2/3 10.8, Q3/4 12.5, and Q4/5 14.8 or (ng/dL) Q1/2 259, Q2/3 311, Q3/4 360, and Q4/5 427; SHBG: (nmol/L) Q1/2 25.8, Q2/3 33.1, Q3/4 40.5, and Q4/5 50.8; cFT: (pmol/L) Q1/2 171, Q2/3 201, Q3/4 230, and Q4/5 268. ^#^Model 1 included terms for testosterone and age and region, with time modelled as a 3-level stratification factor plus an interaction of region with time (see Methods). ^##^Model 2 included model 1 terms + ethnicity (White vs not White), living with partner, educational attainment, alcohol consumption, smoking status, diet (red meat: high vs low vs none), physical activity, BMI, waist circumference, cholesterol, time blood sample collected, blood type, Townsend Index quintile, diabetes, hypertension, angina, atrial fibrillation, COPD, renal impairment, liver disease, thyroid disease, and use of lipid medications (a proxy for hyperlipidemia), glucocorticoids, opioids, and anticonvulsants, with the number of medications included as a proxy for overall comorbidity status. Continuous variables are modelled using restricted cubic splines (see Methods).

**Figure 3:**
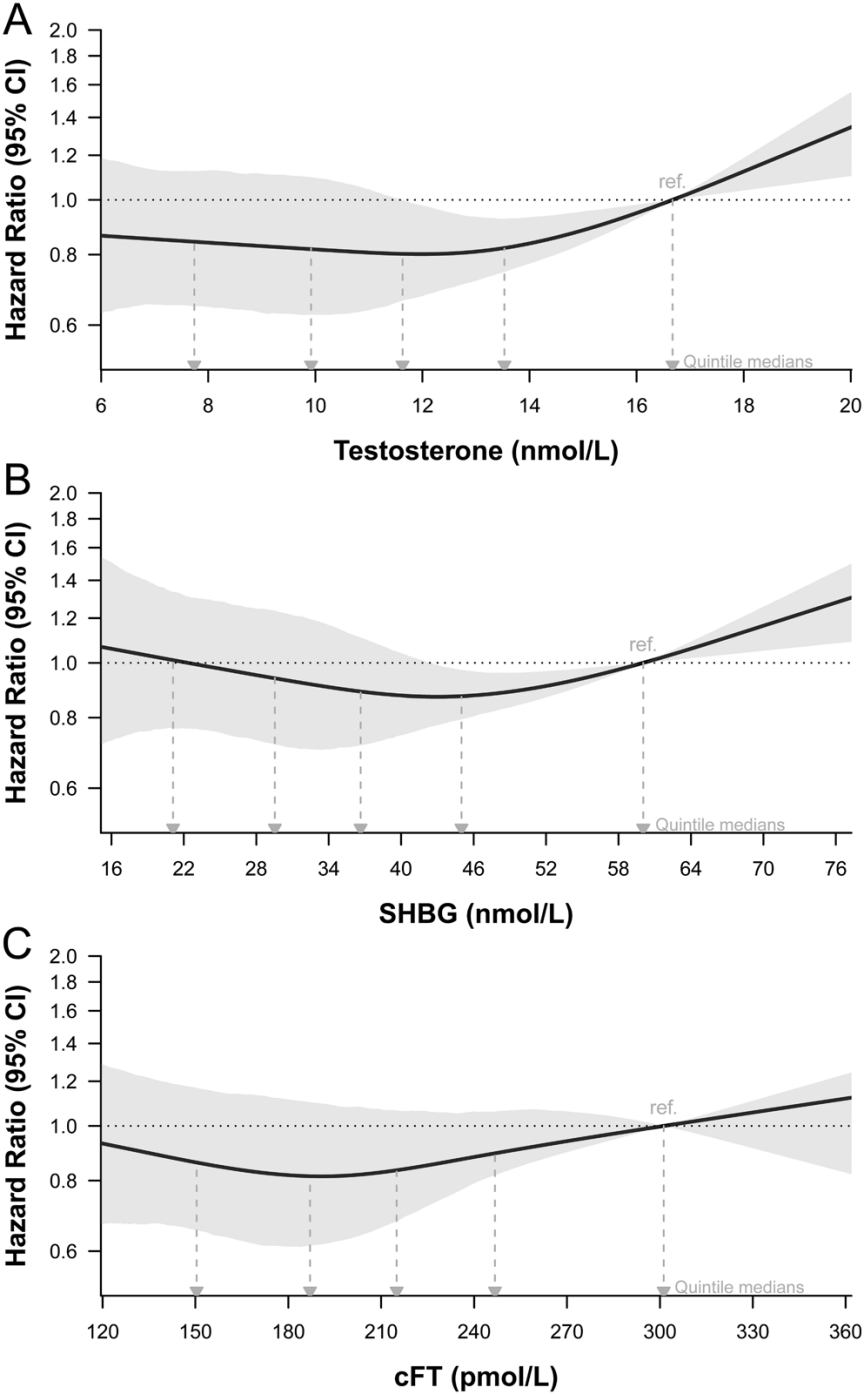
Risk of death from COVID-19, according to baseline serum concentrations of (A) total testosterone, (B) SHBG, and (C) calculated free testosterone (cFT). Model 2: fully adjusted model. Shaded areas are the 95% CIs. Horizontal plot axes are truncated to exclude values lower or higher than the 2.5th and 97.5th percentiles. The location of hazard ratios for medians of quintiles for each exposure variable is shown as they relate to results in Table 3. To convert total testosterone concentrations from nmol/L to mg/dL, divide by 0.0347.

In fully adjusted models which included exposure variable, age, region, and interaction of region with time, the full suite of sociodemographic, lifestyle and medical variables, and time of blood sampling (model 2), total testosterone, and SHBG remained associated with risk of dying from COVID-19 (overall trends, *P* = 0.008 and *P* = 0.008, Table 3). Non-linear relationships were present: men in quintiles 3 and 4 of total testosterone were least likely to die from COVID-19, with 20 and 18% lower risk compared with men in quintile 5 (Fig. 3A and Table 3). However, there was no further reduction in risk for men with total testosterone in quintiles 1 and 2. A non-linear relationship of SHBG with risk of dying from COVID-19 was present in men in quintile 4 of SHBG having a lower risk compared to men in quintile 5 but no further reduction in risk across quintiles 1–3 of SHBG (Fig. 3B and Table 3). Calculated FT was not associated with risk of dying from COVID-19 in the fully adjusted model (Fig. 3C and Table 3).

A sensitivity analysis excluding men with diabetes showed similar results (Supplementary Table 8). In sensitivity analysis for total testosterone with an earlier end-of-follow-up date of 8 December 2020, there were 31.7% fewer COVID-19-related deaths (299 instead of 438). The overall trend was similar, but no longer statistically significant (Supplementary Table 9). In sensitivity analysis for total testosterone with a later end-of-follow-up date of 28 February 2021, a non-linear relationship with COVID-19-related mortality was present, similar to the result of the primary analysis (Supplementary Table 9). There were no substantive differences in other sensitivity analyses for SHBG and cFT (Supplementary Tables 10 and 11).

## Discussion

In community-dwelling men aged 40–69 years, serum total testosterone and SHBG concentrations measured a decade or more prior to the onset of the SARS-CoV-2 pandemic were associated with risk of dying from COVID-19, independently of sociodemographic, lifestyle, and medical factors. The associations were non-linear; men with the highest total testosterone and SHBG concentrations had moderately higher risks of COVID-19-related mortality.

Previous studies have reported lower testosterone concentrations in the setting of men with acute SARS-CoV-2 infections but not their premorbid testosterone concentrations (18, 19, 20, 27, 28). Serum total testosterone was lower in 358 symptomatic men diagnosed with COVID-19 compared with controls, and men with severe COVID-19 had lower testosterone concentrations compared to those with moderate disease (18). In men hospitalised with COVID-19, total testosterone concentrations were lower in those with more severe compared to less severe illness and in those who died compared to eventual survivors (19, 20, 27, 28, 29). Lower serum total testosterone concentrations were associated with more advanced immune activation, characterising more severe and fatal infections (28). Thus, serum total testosterone concentration is a biomarker for acute suppression of the HPT axis during the course of COVID-19, the magnitude of which reflects severity of the acute illness. Variable recovery rates of serum total testosterone concentrations have been reported during follow-up (30, 31, 32).

A novel feature of the present analysis was the examination of premorbid serum total testosterone and SHBG concentrations, measured years prior to any exposure to SARS-CoV-2. As this is a highly infectious virus, being diagnosed with SARS-CoV-2 infection reflects both risk to exposure and susceptibility to infection. In exploratory analyses, common risk factors to exposure (education, ethnicity, Townsend socioeconomic index) were associated with monthly incidence rates of SARS-CoV-2, as were baseline age and BMI. It is possible that some of these sociodemographic factors might have been associated with likelihood of seeking a test. Premorbid total testosterone and SHBG concentrations were not associated with incidence rates, nor were calculated free testosterone values.

Interestingly, premorbid total testosterone concentrations was a predictor of COVID-19 mortality risk. Lower testosterone concentrations in men are associated with higher risk of obesity and type 2 diabetes and with the presence of medical comorbidities, all of which are risk factors for poorer outcomes in COVID-19 (5, 6, 7, 8, 9). The lower limb of the U-shaped relationship between serum total testosterone and COVID-19 mortality risk in minimally adjusted analysis may reflect the association of lower serum testosterone with adverse sociodemographic, lifestyle, and medical factors (11).

In fully adjusted analyses, men with the highest premorbid total testosterone concentrations were at greater risk of dying from COVID-19. The association of SHBG with COVID-19 mortality risk was similar to that of total testosterone, reflecting the close correlation between these two variables (11). Calculation of free testosterone from total testosterone and SHBG concentrations did not provide any further information. In a sensitivity analysis with an earlier end-of-follow-up date, the result for total testosterone was not statistically significant, likely due to the reduced number of outcome events and loss of power. However, the trend was similar to the primary analysis, which was supported by the results of sensitivity analysis with a later end-of-follow-up date.

Total testosterone concentrations were independently associated with forced expiratory volume in 1 s (FEV1) and forced vital capacity (FVC) in community-dwelling men with average age of 50 years (14). Similar findings were reported in a subset of UK Biobank men (15). Testosterone is the classical anabolic steroid, and older men with higher testosterone concentrations are less likely to be or to become frail (33). Despite these potential advantages, men with higher premorbid testosterone concentrations were not protected against COVID-19 mortality risk.

SARS-CoV-2 enters cells via binding of the viral spike protein to ACE2 acting as a cellular receptor, a process which requires priming of the spike protein by host cell proteases (2, 17, 34). The mucosa-specific serine protease TMPRSS2 plays a key role in spike protein priming, facilitating SARS-CoV-2 entry (34). Androgens appear to increase the expression of TMPRSS2, and androgen receptor signalling may modulate ACE2 expression in tissues including lung, providing plausible mechanisms by which higher circulating testosterone concentrations might enhance the entry of SARS-CoV-2 leading to more severe infection (17, 35, 36, 37). Furthermore, higher testosterone concentrations may have immunomodulatory effects contributing to sex differences in interferon responses, function of T and B lymphocytes and other immune cells, and antibody responses, which could potentially worsen outcomes in COVID-19 (2, 16). These pathways might explain our observation that men with the highest premorbid testosterone concentrations, free of confounding from the effects of acute infection on the HPT axis, were at greater risk of dying from COVID-19.

In a study of 80 men hospitalised for COVID-19 randomised to finasteride added to routine treatment vs routine treatment alone, fewer finasteride-treated men died, albeit the results were not statistically significant (1/40 vs 4/40 men) (38). The COVIDENZA trial randomised women and men hospitalized with COVID-19 to enzalutamide and standard care (*n* = 30) vs standard care alone (*n* = 12) and was stopped early due to longer hospitalisations in the active arm (39). In a trial of 268 male outpatients with COVID-19 randomised 1:1 to proxalutamide vs placebo, active treatment reduced the 30-day hospitalisation rate (2.2% vs 26%, *P* < 0.001) (40). Another trial randomised 423 women and men hospitalised with COVID-19 to proxalutamide and 355 to placebo, finding a higher 14-day recovery rate (81.1% vs 36.6%, *P* < 0.001) and lower 14-day all-cause mortality (8.0% vs 39.2%, *P* < 0.001) with active treatment, with similar results at 28 days (41). Our results support further investigation into the possible role of androgen blockade therapy in men with SARS-CoV-2 infection, potentially targeting the window of time before severe acute illness suppresses HPT axis function.

The ability of testosterone to transactivate the androgen receptor in tissues is influenced by the presence of a CAG trinucleotide repeat sequence which encodes a variable-length polyglutamine tract in the receptor (42). Men with shorter CAG trinucleotide repeat sequences, corresponding to androgen receptors with greater transactivation potential, appear to be protected from severe COVID-19 (43, 44). Therefore, genetic polymorphisms may modulate the action of circulating testosterone on outcomes such as COVID-19 severity. Experimental studies suggest that sex hormones may also modulate ACE2 expression and activity, immune cell responses, and coagulation factors (for detailed review, see (45)). The role of sex hormones in relation to more recently identified candidate SARS-CoV-2 receptors such as the tyrosine-protein kinase receptor AXL remains to be elucidated (46, 47).

In the UK Biobank male population, higher SHBG was associated with higher all-cause mortality and with higher dementia risk (21, 48). Our findings that higher SHBG was associated with COVID-19-related mortality are consistent with higher SHBG being a biomarker for several poorer health outcomes in men.

Strengths of the present analysis include the size of UK Biobank, providing a large population-based sample of community-dwelling men with minimal loss to follow-up. The measurement of premorbid total testosterone and SHBG concentrations years prior to any exposure to SARS-CoV-2 avoided confounding from the effects of acute infection on the HPT axis. UK Biobank received results of microbiologically confirmed infection with COVID-19 (26). We included an interaction term of spatial unit with time and adjusted for multiple sociodemographic, lifestyle, and medical factors.

Limitations include the observational nature of the study, precluding determination of causality. Many variables were assessed at baseline by self-report. Mortality outcomes were obtained by data linkage utilising COVID-19 containing ICD codes. Residual confounding from unmeasured variables is possible, even though the fully adjusted model included a range of potentially relevant factors. In the UK Biobank, blood samples were collected throughout the day, thus time of blood sampling was included as a variable in the fully adjusted model. Total testosterone and SHBG were measured using an immunoassay which may underestimate testosterone concentrations compared with mass spectrometry (49). Therefore, we analysed testosterone, SHBG, and cFT as continuous variables, and also in quintiles, enabling the comparison of men across the prevailing range of hormone concentrations and avoiding the use of specific threshold values. This analysis utilised baseline testosterone and SHBG results, and serial measurements leading up to the onset of the pandemic were not available. However, in a subset of UK Biobank men with repeat blood sampling after an interval of 4.3 years, mean serum total testosterone concentrations were stable over time with high concordance between baseline and follow-up results (50). Modelling the effects of vaccination rates, subsequent waves of infection and genetic polymorphisms was outside the scope of this analysis. UK Biobank included men aged 40–69 years, so our findings neither apply to younger or older men nor to men in other regions.

In conclusion, total testosterone and SHBG concentrations were non-linearly associated with COVID-19-related mortality risk in middle-aged to older men. Men with the highest concentrations of total testosterone or SHBG were most at risk, after controlling for age, sociodemographic, lifestyle, and medical factors. Men could be informed that having relatively high serum testosterone concentrations does not protect against future COVID-19-related mortality and encouraged to engage in appropriate measures to reduce their risk of severe SARS-CoV-2 infection. Further investigation of potential underlying mechanisms by which testosterone might impact the severity of SARS-CoV-2 infection is warranted.

## Supplementary materials

Supplementary Material

## Declaration of interest

The authors declare that there is no conflict of interest that could be perceived as prejudicing the impartiality of the research reported.

## Funding

This work was supported by the Western Australian Health Translation Network and the Australian Government’s Medical Research Future Fund as part of the Rapid Applied Research Translation program, a Government of Western Australia Department of Health Future Health and Research Innovation Focus grant, and a philanthropic donation to the University of Western Australia by Lawley Pharmaceuticals, Western Australia. The funding sources had no role in conducting the study, interpreting results, or writing the manuscript.

## Data availability

Data from the UK Biobank are accessible to researchers via application to the UK Biobank.

## References

[1] Tenforde MW Self WH Adams K Gaglani M Ginde AA McNeal T Ghamande S Douin DJ Talbot HK Casey JD Association between mRNA vaccination and COVID-19 hospitalization and disease severity. JAMA 2021 326 2043–2054. (10.1001/jama.2021.19499) 34734975 PMC8569602

[2] Wray S Arrowsmith S. The physiological mechanisms of the sex-based difference in outcomes of COVID19 infection. Frontiers in Physiology 2021 12 627260. (10.3389/fphys.2021.627260) PMC7900431 33633588

[3] Williamson EJ Walker AJ Bhaskaran K Bacon S Bates C Morton CE Curtis HJ Mehrkar A Evans D Inglesby P OpenSAFELY: factors associated with COVID-19 death in 17 million patients. Nature 2020 584 430–436. (10.1038/s41586-020-2521-4) 32640463 PMC7611074

[4] Fortunato F Martinelli D Lo Caputo S Santantonio T Dattoli V Lopalco PL Prato R. Sex and gender differences in COVID-19: an Italian local register-based study. BMJ Open 2021 11 e051506. (10.1136/bmjopen-2021-051506) PMC8507404 34620662

[5] Grasselli G Greco M Zanella A Albano G Antonelli M Bellani G Bonanomi E Cabrini L Carlesso E Castelli G Risk factors associated with mortality among patients with COVID-19 in intensive care units in Lombardy, Italy. JAMA Internal Medicine 2020 180 1345–1355. (10.1001/jamainternmed.2020.3539) 32667669 PMC7364371

[6] Kim L Garg S O’Halloran A Whitaker M Pham H Anderson EJ Armistead I Bennett NM Billing L Como-Sabetti K Risk factors for intensive care unit admission and in-hospital mortality among hospitalized adults identified through the US coronavirus disease 2019 (COVID-19)-associated hospitalization surveillance network (COVID-NET). Clinical Infectious Diseases 2021 72 e206–e214. (10.1093/cid/ciaa1012) 32674114 PMC7454425

[7] Rottoli M Bernante P Belvedere A Balsamo F Garelli S Giannella M Cascavilla A Tedeschi S Ianniruberto S Del Turco ER How important is obesity as a risk factor for respiratory failure, intensive care admission and death in hospitalised COVID-19 patients? Results from a single Italian centre. European Journal of Endocrinology 2020 183 389–397. (10.1530/EJE-20-0541) 32674071 PMC9494325

[8] Tramunt B Smati S Coudol S Wargny M Pichelin M Guyomarch B Al-Salameh A Amadou C Barroud S Bigot E Sex disparities in COVID-19 outcomes of inpatients with diabetes: insights from the CORONADO study. European Journal of Endocrinology 2021 185 299–311. (10.1530/EJE-21-0068) 34085949 PMC9494335

[9] Corona G Pizzocaro A Vena W Rastrelli G Semeraro F Isidori AM Pivonello R Salonia A Sforza A Maggi M. Diabetes is most important cause for mortality in COVID-19 hospitalized patients: systematic review and meta-analysis. Reviews in Endocrine and Metabolic Disorders 2021 22 275–296. (10.1007/s11154-021-09630-8) 33616801 PMC7899074

[10] Feldman HA Longcope C Derby CA Johannes CB Araujo AB Coviello AD Bremner WJ McKinlay JB. Age trends in the level of serum testosterone and other hormones in middle-aged men: longitudinal results from the Massachusetts Male Aging Study. Journal of Clinical Endocrinology and Metabolism 2002 87 589–598. (10.1210/jcem.87.2.8201) 11836290

[11] Yeap BB Marriott RJ Antonio L Bhasin S Dobs AS Dwivedi G Flicker L Matsumoto AM Ohlsson C Orwoll ES Sociodemographic, lifestyle and medical influences on serum testosterone and sex hormone-binding globulin in men from UK Biobank. Clinical Endocrinology 2021 94 290–302. (10.1111/cen.14342) 32979890

[12] Camacho EM Huhtaniemi IT O’Neill TW Finn JD Pye SR Lee DM Tajar A Bartfai G Boonen S Casanueva FF Age-associated changes in hypothalamic-pituitary-testicular function in middle-aged and older men are modified by weight change and lifestyle factors: longitudinal results from the European Male Ageing Study. European Journal of Endocrinology 2013 168 445–455. (10.1530/EJE-12-0890) 23425925

[13] Wittert G Bracken K Robledo KP Grossmann M Yeap BB Handelsman DJ Stuckey B Conway A Inder W McLachlan R Testosterone treatment to prevent or revert type 2 diabetes in men enrolled in a lifestyle programme (T4DM): a randomised, double-blind, placebo-controlled, 2-year phase 3b trial. Lancet 2021 9 32–45. (10.1016/S2213-8587(2030367-3) 33338415

[14] Mohan SS Knuiman MW Divitini ML James AL Musk AW Handelsman DJ Beilin J Hunter M Yeap BB. Higher serum testosterone and dihydrotestosterone, but not oestradiol, are independently associated with favourable indices of lung function in community-dwelling men. Clinical Endocrinology 2015 83 268–276. (10.1111/cen.12738) 25660119

[15] Lenoir A Fuertes E Gomez-Real F Leynaert B van der Plaat DA Jarvis D. Lung function changes over 8 years and testosterone markers in both sexes: UK Biobank. ERJ Open Research 2020 6 00070–2020. (10.1183/23120541.00070-2020) 33015143 PMC7520167

[16] Bunders MJ Altfeld M. Implications of sex differences in immunity for SARS-CoV-2 pathogenesis and design of therapeutic interventions. Immunity 2020 53 487–495. (10.1016/j.immuni.2020.08.003) 32853545 PMC7430299

[17] Wambier CG Goren A Vano-Galvan S Ramos PM Ossimetha A Nau G Herrera S McCoy J. Androgen sensitivity gateway to COVID-19 disease severity. Drug Development Research 2020 81 771–776. (10.1002/ddr.21688) 32412125 PMC7273095

[18] Cinislioglu AE Cinislioglu N Demirdogen SO Sam E Akkas F Altay MS Utlu M Sen IA Yildirim F Kartal S The relationship of serum testosterone levels with the clinical course and prognosis of COVID-19 disease in male patients: a prospective study. Andrology 2022 10 24–33. (10.1111/andr.13081) 34288536 PMC8444851

[19] Rastrelli G Di Stasi V Inglese F Beccaria M Garuti M Di Costanzo D Spreafico F Greco GF Cervi G Pecoriello A Low testosterone levels predict clinical adverse outcomes in SARS-CoV-2 pneumonia patients. Andrology 2021 9 88–98. (10.1111/andr.12821) 32436355 PMC7280645

[20] Camici M Zuppi P Lorenzini P Scarnecchia L Pinnetti C Cicalini S Nicastri E Petrosillo N Palmieri F D’Offizi G Role of testosterone in SARS-CoV-2 infection: a key pathogenic factor and a biomarker for severe pneumonia. International Journal of Infectious Diseases 2021 108 244–251. (10.1016/j.ijid.2021.05.042) 34023492 PMC8135187

[21] Yeap BB Marriott RJ Antonio L Chan YX Raj S Dwivedi G Reid CM Anawalt BD Bhasin S Dobs AS Serum testosterone is inversely, and sex hormone-binding globulin directly, associated with all-cause mortality in men. Journal of Clinical Endocrinology and Metabolism 2021 106 e625–e637. (10.1210/clinem/dgaa743) 33059368

[22] Ly LP Sartorius G Hull L Leung A Swerdloff RS Wang C Handelsman DJ. Accuracy of calculated free testosterone formulae in men. Clinical Endocrinology 2010 73 382–388. (10.1111/j.1365-2265.2010.03804.x) 20346001

[23] Sudlow C Gallacher J Allen N Beral V Burton P Danesh J Downey P Elliott P Green J Landray M UK Biobank: an open access resource for identifying the causes of a wide range of complex diseases of middle and old age. PLoS Medicine 2015 12 e1001779. (10.1371/journal.pmed.1001779) PMC4380465 25826379

[24] Fry D Almond R Moffat S Gordon M Singh P. UK Biobank Biomarker Project. Companion Document to Accompany Serum Biomarker Data, Version 1·0, date 11 March 2019, pp. 1–16. (available at: https://biobank.ndph.ox.ac.uk/showcase/showcase/docs/serum_biochemistry.pdf). Accessed on 25 January 2022.

[25] UK Biobank. Biomarker Assay Quality Procedures: Approaches Used to Minimise Systematic and Random Errors (and the Wider Epidemiological Implications), Version 1.2, date 2 April 2019, pp. 1–15. (available at: https://biobank.ctsu.ox.ac.uk/crystal/crystal/docs/biomarker_issues.pdf). Accessed on 25 January 2022.

[26] Armstrong J Rudkin JK Allen N Crook DW Wilson DJ Wyllie DH O’Connell AM. Dynamic linkage of COVID-19 test results between Public Health England’s Second Generation Surveillance System and UK Biobank. Microbial Genomics 2020 6 mgen000397. (10.1099/mgen.0.000397) PMC7478634 32553051

[27] Cayan S Uguz M Saylam B Akbay E. Effect of serum total testosterone and its relationship with other laboratory parameters on the prognosis of coronavirus disease 2019 (COVID-19) in SARS-CoV-2 infected male patients: a cohort study. Aging Male 2020 23 1493–1503. (10.1080/13685538.2020.1807930) 32883151

[28] Lanser L Burkert FR Thommes L Egger A Hoermann G Kaser S Pinggera GM Anliker M Griesmacher A Weiss G Testosterone deficiency is a risk factor for severe COVID-19. Frontiers in Endocrinology 2021 12 694083. (10.3389/fendo.2021.694083) PMC8253686 34226825

[29] Van Zeggeren IE Boelen A van de Beek D Heijboer AC Vlaar APJ Brouwer MC & Amsterdam UMC COVID-19 Biobank. Sex steroid hormones are associated with mortality in COVID-19 patients: level of sex hormones in severe COVID-19. Medicine 2021 100 e27072. (10.1097/MD.0000000000027072) PMC8389969 34449505

[30] Xu H Wang Z Feng C Yu W Chen Y Zeng X Liu C. Effects of SARS-CoV-2 infection on male sex hormones in recovering patients. Andrology 2021 9 107–114. (10.1111/andr.12942) 33152165

[31] Moreno-Perez O Merino E Alfayate R Torregrosa ME Andres M Leon-Ramirez JM Boix V Gil J Pico A & COVID19-ALC Research group. Male pituitary-gonadal axis dysfunction in post-acute COVID-19 syndrome – prevalence and associated factors: a Mediterranean case series. Clinical Endocrinology 2022 96 353–362. (10.1111/cen.14537) 34160836 PMC8444731

[32] Salonia A Pontillo M Capogrosso P Gregori S Carenzi C Ferrara AM Rowe I Boeri L Larcher A Ramirez GA Testosterone in males with COVID-19: a 7-month cohort study. Andrology 2022 10 34–41. (10.1111/andr.13097) 34409772 PMC8444879

[33] Hyde Z Flicker L Almeida OP Hankey GJ McCaul KA Chubb SAP Yeap BB. Low free testosterone predicts frailty in older men. The Health in Men Study. Journal of Clinical Endocrinology and Metabolism 2010 95 3165–3172. (10.1210/jc.2009-2754) 20410223

[34] Hoffmann M Kleine-Weber H Schroeder S Kruger N Herrier T Erichsen S Schiergens TS Herrier G Wu NH Nitsche A SARS-CoV-2 cell entry depends on ACE2 and TMPRSS2 and is blocked by a clinically proven protease inhibitor. Cell 2020 181 271.e8–280.e8. (10.1016/j.cell.2020.02.052) 32142651 PMC7102627

[35] Samuel RM Majd H Richter MN Ghazizadeh Z Zekevat SM Navickas A Ramirez JT Asgharian H Simoneau CR Bonser LR androgen signalling regulates SARS-CoV-2 receptor levels and is associated with severe COVID-19 symptoms in men. Cell Stem Cell 2020 27 876.e12–889.e12. (10.1016/j.stem.2020.11.009) 33232663 PMC7670929

[36] Qiao Y Wang XM Mannan R Pitchiaya S Zhang Y Wotring JW Xiao L Robinson DR Wu YM Tien JC-Y Targeting transcriptional regulation of SARS-CoV-2 entry factors *ACE2* and *TMPRSS2*. PNAS 2020 118 e2021450118. (10.1073/pnas.2021450118) PMC7817128 33310900

[37] Chanana N Palmo T Sharma K Kumar R Graham BB Pasha Q. Sex-derived attributes contributing to SARS-CoV-2 mortality. American Journal of Physiology: Endocrinology and Metabolism 2020 319 E562–E567. (10.1152/ajpendo.00295.2020) 32726128 PMC7473885

[38] Zarehoseinzade E Allami A Ahmadi M Bijani B Mohammadi N. Finasteride in hospitalized adult males with COVID-19: a risk factor for severity of the disease or an adjunct treatment: a randomized controlled clinical trial. Medical Journal of the Islamic Republic of Iran 2021 35 30. (10.47176/mjiri.35.30) PMC8214036 34169042

[39] Welen K Rosendal E Gisslen M Lenman A Freyhult E Fonseca-Rodriguez O Bremell D Stranne J Balkhed ÅÖ Niward K A phase 2 trial of the effect of antiandrogen therapy on COVID-19 outcome: no evidence of benefit, supported by epidemiology and in vitro data. European Urology 2022 81 285–293. (10.1016/j.eururo.2021.12.013) 34980495 PMC8673828

[40] McCoy J Goren A Cadegiani FA Vano-Galvin S Kovacevic M Situm M Shapiro J Sinclair R Tosti A Stanimirovic A Proxalutamide reduces the rate of hospitalization for COVID-19 male outpatients: a randomized double-blinded placebo-controlled trial. Frontiers in Medicine 2021 8 668698. (10.3389/fmed.2021.668698) PMC8326462 34350193

[41] Cadegiani FA Zimerman RA Fonseca DN Correia MN Muller MP Bet DL Slaviero MR Zardo I Benites PR Barros RN Final results of a randomized, placebo-controlled, two-arm, parallel clinical trial of proxalutamide for hospitalized COVID-19 patients: a multiregional, joint analysis of the Proxa-Rescue AndroCoV Trial. Cureus 2021 13 e20691. (10.7759/cureus.20691) PMC8712234 34976549

[42] Simanainen U Brogley M Gao YR Jimenez M Harwood DT Handelsman DJ Robins DM. Length of the human androgen receptor glutamine tract determines androgen sensitivity in vivo. Molecular and Cellular Endocrinology 2011 342 81–86. (10.1016/j.mce.2011.05.011) 21664242 PMC3148310

[43] Baldassarri M Pichiotti N Fava F Fallerini C Benetti E Daga S Valentino F Doddato G Furini S Giliberti A Shorter androgen receptor polyQ alleles protect against life-threatening COVID-19 disease in European males. EBiomedicine 2021 65 103246. (10.1016/j.ebiom.2021.103246) PMC7908850 33647767

[44] McCoy J Wambier CG Herrera S Vano-Galvan S Gioia F Comeche B Ron R Serrano-Villar S Iwasiou RM Tayeb MA Androgen receptor genetic variant predicts COVID-19 disease severity: a prospective longitudinal study of hospitalized COVID-19 male patients. Journal of the European Academy of Dermatology and Venereology 2021 35 e15–e17. (10.1111/jdv.16956) 32977355 PMC7536899

[45] Pivonello R Auriemma RS Pivonello C Isidori AM Corona G Colao A Millar RP. Sex disparities in COVID-19 severity and outcome: are men weaker or women stronger? Neuroendocrinology 2021 111 1066–1085. (10.1159/000513346) 33242856 PMC7900484

[46] Bohan D Van Ert H Ruggio N Rogers KJ Badreddine M Aguilar Briseño JA Elliff JM Rojas Chavez RA Gao B Stokowy T Phosphatidylserine receptors enhance SARS-CoV-2 infection. PLoS Pathogens 2021 17 e1009743. (10.1371/journal.ppat.1009743) PMC8641883 34797899

[47] Wang S Qiu Z Hou Y Deng X Xu W Zheng T Wu P Xie S Bian W Zhang C AXL is a candidate receptor for SARS-CoV-2 that promotes infection of pulmonary and bronchial epithelial cells. Cell Research 2021 31 126–140. (10.1038/s41422-020-00460-y) 33420426 PMC7791157

[48] Marriott RJ Murray K Flicker L Hankey GJ Matsumoto AM Dwivedi G Antonio L Almeida OP Bhasin S Dobs AS Lower serum testosterone concentrations are associated with a higher incidence of dementia in men: the UK Biobank prospective cohort study. Alzheimer’s and Dementia 2022 In Press. (10.1002/alz.12529) 34978125

[49] Dittadi R Matteucci M Meneghetti E Ndreu R. Reassessment of the access testosterone chemiluminescence assay and comparison with the LC-MS method. Journal of Clinical Laboratory Analysis 2018 32 e22286. (10.1002/jcla.22286) PMC6817188 28643405

[50] Marriott RJ Murray K Hankey GJ Manning L Dwivedi G Wu FCW Yeap BB. Longitudinal changes in serum testosterone and sex hormone-binding globulin in men aged 40–69 years from the UK Biobank. Clinical Endocrinology 2022 96 589–598. (10.1111/cen.14648) 34873743

