# Supplementary material for: Higher premorbid serum testosterone predicts COVID-19-related mortality risk in men
Source: Eur J Endocrinol. 2022 May 10;187(1):159–70. doi: 10.1530/EJE-22-0104 (PMC9175556; doi:10.1530/EJE-22-0104)

Supplemental data

Higher premorbid serum testosterone predicts COVID-19-related mortality risk in men.

Authors:

Bu B. Yeap, Ross J. Marriott, Laurens Manning, Girish Dwivedi, Graeme J. Hankey, Frederick C.W. Wu, Jeremy K. Nicholson, Kevin Murray

Supplemental Methods

Variables of interest

Social, demographic, and lifestyle variables

Educational attainment (qualifications) was categorised as below A-levels (high school), completed A-levels, completed college/university, or completed other professional qualification (not school/college/university). Townsend Deprivation Index scores were grouped into quintile categories, with Quintile 5 being the most disadvantaged.[1] Alcohol consumption was categorised using a simplification of categories used by Topiwala et al.: "low" (<14 units/week) including abstainers and men with safe levels of consumption, consistent with UK guidelines; "medium" (14-<30 units/week); "high" ≥30 units/week (1 unit=8 g pure alcohol).[2,3] Diet categories were high red meat (beef, lamb and pork >3 times/week), low red meat (≤3/week), no red meat, which is a simplification of Bradbury et al.[4] Ethnicity categories were White or Not white (includes Asian, Black, Chinese, Mixed, Other). Vigorous and moderate physical activity undertaken per week was categorised to World Health Organization recommendations as sufficient: ≥75 minutes vigorous or ≥150 minutes moderate, or equivalent combination; insufficient: less than this; additional: ≥150 minutes vigorous- or ≥300 minutes moderate-level or equivalent combination (for "additional" health benefits).[5]

Assessment centres were grouped into geographical regions of the UK following Lin et al. for analyses of COVID-19 deaths (South West, South East, London, East Midlands, West Midlands, Yorkshire & The Humber, North East, North West, Scotland, Wales).[6] Assessment centres were grouped into coarser geographical units of country (England, Scotland, Wales) for analyses of SARS-CoV-2 infection rates.

Blood biomarkers

Blood type (A, AB, B, O) was calculated from the UK Biobank blood group variable. Cholesterol (mmol/L) was measured from blood samples by CHO-POD analysis on a Beckman Coulter AU5800.[7]

Prevalent medical conditions

Prevalent medical conditions at baseline were determined using International Classification of Diseases (ICD) diagnosis codes from previous hospital inpatient admissions, recorded across all records in either the primary or secondary diagnoses positions, or self-reported data. A history of cancer diagnosis was determined using cancer registry and self-reported data. Prevalent diabetes was determined using hospital inpatient diagnoses, self-reported diabetes, self-reported insulin use, blood glucose (fasting time missing or <8 hr) ≥11·1 mmol/L, fasting (≥8 hr) blood glucose ≥7·0 mmol/L, or HbA1c ≥48 mmol/mol. Prevalent hypertension was determined using hospital inpatient diagnoses, self-reported high blood pressure, self-reported usage of blood pressure medications, or a measured systolic blood pressure ≥140 mmHg, or diastolic blood pressure ≥90 mmHg. Prevalent renal impairment was determined using hospital inpatient diagnoses, self-reported data, or serum creatinine >150 umol/L. Prevalent dementia, which was determined using hospital inpatient diagnoses,

self-reported data, or self-reported use of anti-dementia medications, and HIV were initially considered for inclusion in analyses but were not used due to very low or zero numbers of events for participants with these conditions at baseline. Further information and ICD codes are provided in Supplemental Table S2. Medication usage (number of medications, lipid medication, anticonvulsant, glucocorticoid, opioid use) was identified from verbal interviews of participants at baseline on their use of prescription and over-the-counter medicines (UK Biobank Variable 20003). Number of medications was recoded into categories of 0, 1-2, 3-4, 5+ medications taken, consistent with recent NHS reporting.[8,9]

Statistical analysis

Overview.

There were two sets of analyses conducted: (i) an exploratory investigation of possible associations of endogenous hormone concentrations in UK Biobank men with incidence rate of SARS-CoV-2 infections; (ii) to investigate possible associations of endogenous hormone concentrations in UK Biobank men with incident risk of death from COVID-19. Endogenous hormone concentrations were measured at baseline (2006-2010), with a focus on total testosterone, although analyses were replicated for SHBG, and calculated free testosterone (cFT), as additional exposures of interest.

Occurrence of infections across regions

Preliminary inspection of a heat map of the reported numbers of incident infections for the whole cohort (males and females combined) showed that the pattern of occurrence (infections) varied spatially during follow-up (Supplemental Figure S1). Accordingly, all analyses included an interaction term of spatial unit with time.

Associations of testosterone and SHBG with incidence of SARS-CoV-2 infection

Monthly incidence rates of SARS-CoV-2 infections (per 1000 person-months) were calculated by dividing the number of incident events in that month by the number at risk at the start of that month, then multiplying by 1000. For use in Figure 1 these were calculated using:

$$IR_{h,m} = 1000 \times \frac{Count_{h,m}}{At\ Risk_{h,m}}$$

where *IR* = incidence rate, *h* = hormone quintile, *m* = month, *Count* = number of participants with an incident infection, *At Risk* = number alive and still at risk at the start of that month.

For interpretability of Incidence Rate Ratios (IRRs), model predictors were modelled as categories in multivariable analyses of incidence rates, with the IR calculated as:

$$IR_{h,a,b,c,d,e,r,m} = 1000 \times \frac{Count_{h,a,b,c,d,e,r,m}}{At\ Risk_{h,a,b,c,d,e,r,m}}$$

where *a* = baseline age category (≤50, 51-60, >60 years), *b* = baseline BMI (<25, 25-<30, ≥30 kg/m^2), *c* = ethnicity (white, not white), *d* = educational attainment/qualifications (not completed college or university, college or university), *e* = Townsend Index quintile, *h* = baseline hormone (total testosterone, SHBG, or cFT) concentration modelled as quintile categories, *r* = Country (England, Scotland, Wales). Month was modelled using a natural cubic spline with a knot point set at the day preceding the introduction of the 'Rule of 6' social distancing measure (14 September 2020), the first of a series of national restrictions introduced to address the second wave of the epidemic. Zero-Inflated Poisson regressions were fitted to account for an excess of zero frequencies in the counts (response variable), with the logged number at risk modelled as an offset term.

Vaccination availability in the United Kingdom

Although vaccination data were not available for this cohort, 17.6 % and 0.9% of the UK population had been vaccinated once and twice by 31 January 2021, respectively (Supplemental Table S1).

Sensitivity analyses were conducted to evaluate the use of two alternative end of follow-up dates: (i) 8 December 2021, when vaccinations commenced in the UK; and (ii) 28 February 2021, which occurred within the tail of the second wave and, at the time of analysis, was the latest date to which death and cause of death data were complete. Graphical inspection of data for sensitivity analysis (i) identified a steep decline in incidence rate from November to December, which was an artefact of the specification of 8 December as the end of follow-up date, and thus a relatively short amount of exposure time for incident events to have occurred in that month. Therefore, to avoid the possible influence of this setting on estimated coefficients for Month, December data (count of incident infections and number at risk) were excluded from sensitivity analysis (i).

Associations of testosterone and SHBG with COVID-19 deaths

A Cox proportional hazards model, including an interaction term of time stratum (Wave 1, 16 March 2020 – 13 September 2020; Wave 2, 14 September 2020 – 31 January 2021) with UK Region (South West, South East, London, East Midlands, West Midlands, Yorkshire & The Humber, North East, North West, Scotland, Wales) was initially fitted to time-to-covid-19 death data. A minimally adjusted model (Model 1) included the exposure variable, baseline age, UK region and the interaction term. Model 2 included additional covariates: lifestyle and demographic variables (ethnicity, living with partner, alcohol consumption, diet, physical activity, educational attainment, smoking status, waist circumference, BMI, cholesterol), time of day for blood sampling (decimal hours), blood type, blood cholesterol, prevalent condition

variables (history of angina, atrial fibrillation, cancer, cardiovascular disease, chronic obstructive pulmonary disease, diabetes, hypertension, liver disease, renal impairment, thyroid disease), and prevalent medication usage variables (anticonvulsants, lipid, glucocorticoids, opioids, number of medications used). Continuous predictors, including that for the exposure in each analysis (total testosterone, SHBG, cFT), were modelled using restricted cubic splines with boundary knots set at the 5th and 95th percentiles and inner knots at the 35th and 65th percentiles.[10]

The validity of the proportional hazards assumption was assessed using per-variable and global tests.[11] Plots of the Schoenfeld residuals, with estimated coefficients and 95% confidence intervals plotted against follow-up times, were inspected for statistically significant results. Test results were ignored when the detected temporal variation for proportional hazard estimates was shown to be negligible.[12] Violation of the proportional hazards assumption for the interaction term of UK region with time stratum was resolved by refitting these models instead with three time strata (Wave 1, 16 March 2020 - 13 September 2020; Wave 2 pre-vaccinations, 14 September 2020 - 7 December 2020; Wave 2 after commencement of vaccinations, 8 December 2020 - 31 January 2021).

Hazard ratios (HRs) and 95% CIs were calculated from each of the fitted models, relative to the median of the fifth sample quintile (the reference value). Percentile bootstrap estimates of 95% CIs were calculated using 2,000 bootstrap iterations. HRs and 95% CIs relative to the reference value of the exposure variable were calculated and plotted against the exposure variable over a continuous range, to show non-linear effects in figures. HRs and 95% CIs associated with the change in hormone concentration from this reference value to the median of each of the other sample quintiles were shown in tables.

As above, we evaluated the sensitivity of fully-adjusted estimates from Model 2 analyses to the use of two alternative end of follow-up dates: (i) 8 December 2021; and (ii) 28 February 2021. Accordingly, for sensitivity analysis (i) the time strata were simplified to Wave 1 (16 March 2020 – 13 September 2020) and Wave 2 pre-vaccinations (14 September 2020 – 8 December 2020). In sensitivity analysis (ii) the extension of the end date from 31 January 2021 to 28 February 2021 led to a violation of the proportional hazards assumption for the interaction term of UK region with time stratum. This was resolved, in part, by adding a fourth time stratum to get: Wave 1 (16 March 2020 – 13 September 2020); Wave 2 pre-vaccinations (14 September 2020 – 7 December 2020); Wave 2 vaccinations (8 December 2020 – 31 January 2021; Extended follow-up (1 – 28 February 2021) time strata. A further violation of the proportional hazards assumption was resolved by adding an interaction term of prevalent liver disease with time stratum, and, for analyses of cFT, an additional interaction of BMI (modelled using a restricted cubic spline) with time stratum.

Supplemental references

1. Hastie CE, Pell JP, Sattar N. Vitamin D and COVID-19 infection and mortality in UK Biobank. Eur J Nutr 2021; 60: 545-8.
2. Topiwala A, Allan CL, Valkanova V, et al. Moderate alcohol consumption as risk factor for adverse brain outcomes and cognitive decline: longitudinal cohort study. BMJ 2017; 357: j2353.
3. UK alcohol unit guidance: CMOs' Low Risk Drinking Guidelines. https://www.drinkaware.co.uk/facts/alcoholic-drinks-and-units/latest-uk-alcohol-unit-guidance (accessed 28 October 2020).

4. Bradbury KE, Tong TYN, Key TJ. Dietary intake of high-protein foods and other major foods in meat-eaters, poultry-eaters, fish-eaters, vegetarians, and vegans in UK Biobank. Nutrients 2017; 9: 1317.
5. Global Recommendations on Physical Activity for Health: 18-64 years old. World Health Organisation, 2011. https://www.who.int/dietphysicalactivity/pa/en/ (accessed 18 February, 2020).
6. Lin L-Y, Smeeth L, Langan S, et al. Distribution of vitamin D status in the UK: a cross-sectional analysis of UK Biobank. BMJ Open 2021; 11: e038503.
7. Fry D, Almond R, Moffat S, Gordon M, Singh P. UK Biobank Biomarker Project. Companion document to accompany serum biomarker data. Version 1.0, date 11 March 2019, 1-16. https://biobank.ndph.ox.ac.uk/showcase/showcase/docs/serum_biochemistry.pdf (accessed 9 September 2021).
8. Zanetti D, Bergman H, Burgess S, Assimes TL, Bhalla V, Ingelsson E. Urinary albumin, sodium, and potassium and cardiovascular outcomes in the UK Biobank: Observation and Mendelian Randomization analyses. Hypertension 2020; 75: 714-22.
9. Moody A, Mindell J, Faulding S. Health Survey for England 2016. Prescribed medicines. NHS Digital. http://healthsurvey.hscic.gov.uk/media/63790/HSE2016-pres-med.pdf (accessed May 5, 2020).
10. Harrell FE Jr. 2015. Regression modelling strategies with applications to linear models, logistic and ordinal regression, and survival analysis. Second Edition. Springer.
11. Grambsch P, Therneau T. Proportional hazards tests and diagnostics based on weighted residuals. Biometrika 1994; 81: 515-26.
12. Therneau TM, Grambsch PM. Modeling survival data: extending the Cox model. Springer-Verlag: New York 2000.

Supplemental Tables

Table S1. Cumulative number of SARS-2-CoV cases, vaccinations, and percentage vaccinated within each country, and for the whole of the United Kingdom at three candidate end of follow-up dates. [Source: https://coronavirus.data.gov.uk, accessed 11 May, 2021].

	England	Wales	Scotland	UK
As at 08/12/2020*:				
No. cases	1,537,627	98,736	103,417	1,795,672
As at 31/01/2021:				
No. Vaccinations (1st)	8,082,355	416,306	575,897	9,296,367
(2nd)	460,907	841	7,849	494,209
% Vaccinated (1st)	18.3	16.5	13.0	17.6
(2nd)	1.0	Not reported	0.2	0.9
No. cases	3,371,490	193,920	181,210	3,850,563
As at 28/02/2021:				
No. Vaccinations (1st)	17,212,804	925,669	1,611,578	20,275,451
(2nd)	599,935	103,819	78,865	815,816
% Vaccinated (1st)	35.6	33.6	33.7	35.3
(2nd)	1.2	3.8	1.6	1.4
No. cases	3,663,892	204,246	202,001	4,181,530

* = Vaccinations in the UK commenced on this date.

Table S2. Hospital diagnosis and death registry codes used to identify incident health conditions of interest or prevalent conditions, for model covariates or exclusions.*

**Health condition**	**ICD-9**	**ICD-10**
Adrenogenital / testicular disorders	255.2, 257	E25, E29
Pituitary disease	253.0-253.4, 253.7	E22.0, E22.1, E22.8, E22.9, E23.0, E22.1, E23.3, E24
Angina	413.0, 413.1, 413.9	I20.0, I20.1, I20.8, I20.9
Atrial Fibrillation	427.3	I48, and, where the 4th digit was provided**, I48.0, I48.1, I48.2, I48.9
COPD	490, 491, 492, 494, 496	J43.1, J43.2, J43.8, J43.9, J44.0, J44.1, J44.8, J44.9
CVD	410, 428, 430-437	I21, I22, I50, I60-I64, I69.0-I69.4
Diabetes	250.0 - 250.9	E10 - E14
Hypertension	401	I10
Liver disease	570-573	K70-K76
Renal Impairment	582, 583, 585, 586	N03, N04, N05, N08.1-NO8.3, NO8.5, N11.1, N11.8, N11.9, N14, N18.2, N18.3, N18.4, N18.5, N18.6, N18.9, N19
Thyroid disease	240-245	E00-E06

* = Additional data sources were also used for identifying prevalent conditions (e.g., from self-report medical conditions, self-report medication usage, physical examination and blood chemistry measurements).

** = In many cases only "I48" was provided but in others the full 4 digit code was provided.

Table S3. Summary statistics for male UK Biobank participants included in analyses (complete cases) as compared to those not included because of missing covariate values (incomplete cases).** The number and percentage of missing values for each variable are also presented.**

Characteristic[§]	**Complete cases*** *(n = 159,964)*	**Incomplete cases*** *(n = 46,758)*	**Missing values** *per variable: n (%)*
Sociodemographic & Lifestyle			
Age (whole years)	57.0 (50.0-63.0)	58.0 (50.0-63.0)	0 (0.0)
BMI (kg/m^2)	27.2 (25.0-29.9)	27.3 (25.0-30.1)	1,345 (0.7)
Waist circumference (cm)	96.0 (89.0-103.0)	96.0 (89.0-104.0)	900 (0.4)
Country: England	88.7 (141,850)	90.8 (42,462)	0 (0.0)
Scotland	7.0 (11,140)	5.9 (2,744)	
Wales	4.4 (6,974)	3.3 (1,552)	
Townsend Index: Q1	20.6 (32,949)	17.6 (8,171)	269 (0.1)
Q2	20.5 (32,717)	18.0 (8,349)	
Q3	20.4 (32,629)	19.1 (8,902)	
Q4	19.7 (31,557)	20.6 (9,555)	
Q5	18.8 (30,112)	24.8 (11,512)	
Ethnicity: Not white	4.8 (7,717)	8.4 (3,807)	1,340 (0.6)
Education: College/University	36.1 (57,680)	29.7 (13,186)	2,302 (1.1)
Partner: True	78.3 (125,271)	73.7 (33,830)	850 (0.4)
Alcohol consumption: Low	40.5 (64,850)	45.7 (21,048)	674 (0.3)
Medium	29.6 (47,376)	27.6 (12,715)	
High	29.8 (47,738)	26.7 (12,321)	
Diet: High Red Meat eaters	16.0 (25,561)	17.1 (7,371)	3,613 (1.7)
Low Red Meat eaters	80.5 (128,826)	79.6 (34,323)	
No Red Meat	3.5 (5,577)	3.4 (1,451)	
Physical Activity: Insufficient	39.9 (63,768)	41.8 (14,518)	12,063 (5.8)
Sufficient	15.9 (25,403)	15.5 (5,363)	
Additional	44.3 (70,793)	42.7 (14,814)	

Characteristic[§]	**Complete cases*** *(n = 159,964)*	**Incomplete cases*** *(n = 46,758)*	**Missing values** *per variable: n (%)*
Smoking: Never	50.7 (81,042)	50.0 (22,769)	1,245 (0.6)
Previous	38.0 (60,770)	36.9 (16,782)	
Current	11.3 (18,152)	13.1 (5,962)	
Prevalent health conditions and medication usage			
CVD	5.3 (8,516)	6.3 (2,965)	0 (0.0)
Diabetes	7.0 (11,208)	8.5 (3,992)	0 (0.0)
Cancer	4.3 (6,866)	4.4 (2,035)	0 (0.0)
Angina	4.9 (7,783)	5.8 (2,728)	0 (0.0)
Atrial Fibrillation	2.0 (3,175)	1.9 (910)	0 (0.0)
Renal impairment	0.6 (916)	0.6 (303)	0 (0.0)
Hypertension	61.9 (99,002)	62.8 (29,342)	0 (0.0)
COPD	0.6 (984)	0.9 (418)	0 (0.0)
Liver disease	1.2 (1,937)	1.5 (680)	0 (0.0)
Thyroid disease	2.1 (3,296)	2.2 (1,048)	0 (0.0)
Lipid medication use	22.4 (35,868)	23.4 (10,830)	407 (0.2)
Glucocorticoid use	7.0 (11,168)	7.0 (3,253)	407 (0.2)
Opioid use	4.0 (6,405)	5.4 (2,502)	407 (0.2)
Anticonvulsant use	1.3 (2,109)	1.8 (825)	407 (0.2)
No. medications: None	33.3 (53,267)	32.5 (15,087)	407 (0.2)
1-2	33.3 (53,337)	31.8 (14,752)	
3-4	18.2 (29,166)	18.4 (8,517)	
5+	15.1 (24,194)	17.2 (7,995)	
Blood / hormone variables			
Time blood drawn (dec hr)	14.5 (11.8-17.0)	14.6 (11.9-17.0)	1,496 (0.7)
Blood type: A	43.2 (69,153)	42.9 (17,852)	5,142 (2.5)
B	9.5 (15,198)	10.3 (4,299)	

Characteristic§	**Complete cases*** *(n = 159,964)*	**Incomplete cases*** *(n = 46,758)*	**Missing values** *per variable: n (%)*
AB	3.6 (5,730)	3.9 (1,606)	
O	43.7 (69,883)	42.9 (17,859)	
Cholesterol (mmol/L)	5.5 (4.8-6.2)	5.4 (4.7-6.2)	12,528 (6.1)
Testosterone (nmol/L)	11.6 (9.5-14.1)	11.7 (9.4-14.2)	14,309 (6.9)
Testosterone (ng/dL)	334 (274-406)	337 (271-409)	14,309 (6.9)
SHBG (nmol/L)	36.6 (27.7-47.6)	36.9 (27.7-48.2)	28,698 (13.9)
cFT (pmol/L)	215 (180-257)	214 (177-257)	29,598 (14.3)

* = Continuous variables (age, BMI, cFT, cholesterol, SHBG, testosterone, time blood drawn, waist circumference) represented as median (interquartile range); other variables as percentages (numbers) per category.
§ = cFT = calculated free testosterone; Smoking, smoking status; CVD, Cardiovascular Disease; SHBG, sex hormone binding globulin.
** After exclusions for prior pituitary disease, orchidectomy, adrenogenital/testicular disorders, or taking androgen, anti-androgen, estrogen, anti-estrogen, progesterone, or 5α-reductase inhibitor medications, or were self-reported infertile.

Table S4. Baseline characteristics of UK Biobank men stratified according to those who were tested for infection, or were infected with or died from COVID-19 during the follow-up period, and for the cohort as a whole**. Summary statistics presented for means and standard deviations of those continuous variables that are presented in Table 1.

	Participants with this event recorded during follow-up			**All****	
Baseline Characteristic* [§]	**Tested** *(n =24,175)*	**Infected with SARS-CoV-2** [§§] *(n=5,558)*	**Died from COVID-19** *(n=438)*	*(n=159,964)*	Skewness
Sociodemographic & Lifestyle					
Age (whole years)	57.0 (8.3)	54.2 (8.8)	63.3 (5.5)	56.2 (8.2)	-0.29
BMI (kg/m^2)	28.3 (4.4)	28.7 (4.4)	29.7 (5.3)	27.8 (4.1)	1.01
Waist circumference (cm)	98.1 (11.6)	98.5 (11.7)	102.8 (13.3)	96.6 (11.1)	0.64
Blood / hormone variables					
Time blood drawn (dec hr)	14.4 (3.0)	14.5 (3.0)	14.3 (2.8)	14.4 (3.0)	-0.03
Cholesterol (mmol/L)	5.4 (1.1)	5.5 (1.1)	5.1 (1.2)	5.5 (1.1)	0.21
Testosterone (nmol/L)	11.8 (3.7)	11.8 (3.7)	11.5 (4.3)	12.0 (3.6)	0.74
Testosterone (ng/dL)	340 (105)	340 (107)	332 (124)	345 (104)	0.74
SHBG (nmol/L)	39.0 (16.4)	36.8 (15.9)	42.7 (20.2)	39.1 (16.2)	1.22
cFT (pmol/L)	218.6 (64.8)	227.1 (71.0)	200.3 (70.9)	221.9 (63.8)	1.52

* = Continuous variables (age, BMI, cholesterol, waist circumference, time blood drawn, testosterone, SHBG, cFT) represented as mean (standard deviation).
** = Summary data presented for data after excluding men who died or were lost to follow-up since their baseline visit but before 16 Mar 2020, with prior orchidectomy, taking androgens, anti-androgen, 5α-reductase, estrogen, anti-estrogen, progesterone medications, infertile men, men with pituitary disease, adrenogenital or testicular disorders, or variables with missing values.
§ = BMI, body mass index (kg/m^2); SHBG, sex hormone binding globulin; cFT, free testosterone calculated using the Vermeulen formula.
§§ = Incident infections identified for participants with a positive test result or who died from COVID-19 during the follow-up period from 16 Mar 2020 to 31 Jan 2021.

Table S5. Results from sensitivity analysis after excluding participants with diabetes: Incidence Rate Ratios (IRRs) and IRR 95% confidence intervals of SARS-CoV-2 infections in UK Biobank men during follow-up (16 March 2020 – 31 January 2021). Estimates presented for model predictors of interest, including for quintile categories of the baseline hormone concentration (Testosterone, SHBG, cFT), from Poisson regression.

	Hormone term modelled as quintile categories*		
**Predictor**	**Testosterone**	**SHBG**	**cFT**
Hormone: Quintile 5 (ref)	1	1	1
Quintile 4	1.02 (0.93-1.12)	1.04 (0.94-1.14)	1.03 (0.95-1.13)
Quintile 3	1.00 (0.91-1.09)	1.00 (0.91-1.11)	0.98 (0.89-1.07)
Quintile 2	0.97 (0.88-1.06)	1.07 (0.97-1.18)	1.01 (0.92-1.11)
Quintile 1	1.01 (0.92-1.11)	1.04 (0.94-1.14)	1.00 (0.90-1.10)
Age: ≤50 (ref)	1	1	1
51-60	0.61 (0.57-0.65)	0.62 (0.58-0.66)	0.61 (0.57-0.66)
>60	0.52 (0.49-0.56)	0.54 (0.50-0.58)	0.53 (0.49-0.57)
BMI: <25 (ref)	1	1	1
25-<30	1.30 (1.20-1.41)	1.29 (1.20-1.40)	1.29 (1.20-1.40)
≥30	1.65 (1.51-1.80)	1.62 (1.48-1.77)	1.65 (1.52-1.80)
Ethnicity: not white	1.60 (1.43-1.79)	1.61 (1.43-1.80)	1.60 (1.43-1.80)
Quals: College/University	0.68 (0.63-0.72)	0.67 (0.63-0.72)	0.68 (0.63-0.72)
Townsend Index: Quintile 1 (ref)	1	1	1
Quintile 2	0.98 (0.89-1.08)	0.98 (0.89-1.08)	0.99 (0.90-1.09)
Quintile 3	1.04 (0.95-1.14)	1.04 (0.95-1.15)	1.03 (0.94-1.14)
Quintile 4	1.15 (1.05-1.26)	1.12 (1.02-1.23)	1.14 (1.04-1.25)
Quintile 5	1.33 (1.21-1.46)	1.32 (1.21-1.45)	1.34 (1.22-1.47)

* Quintile boundaries: Testosterone (nmol/L) Q1/2 9.0, Q2/3 10.8, Q3/4 12.5 and Q4/5 14.8 or (ng/dL) Q1/2 259, Q2/3 311, Q3/4 360 and Q4/5 427; SHBG (nmol/L) Q1/2 25.8, Q2/3 33.1, Q3/4 40.5 and Q4/5 50.8; cFT (pmol/L) Q1/2 171, Q2/3 201, Q3/4 230 and Q4/5 268.

Table S6. Incidence Rate Ratios (IRRs) and IRR 95% confidence intervals of SARS-CoV-2 infections in UK Biobank men during follow-up (16 March 2020 – 8 December 2020*).

	Hormone term modelled as quintile categories*		
**Predictor**	**Testosterone**	**SHBG**	**cFT**
Hormone: Quintile 5 (ref)	1	1	1
Quintile 4	1.02 (0.90-1.16)	1.05 (0.92-1.20)	1.03 (0.92-1.16)
Quintile 3	0.98 (0.86-1.11)	1.04 (0.91-1.18)	0.93 (0.82-1.05)
Quintile 2	1.00 (0.88-1.13)	1.01 (0.88-1.15)	1.02 (0.90-1.15)
Quintile 1	0.98 (0.87-1.12)	1.04 (0.91-1.18)	0.97 (0.85-1.10)
Age: ≤50 (ref)	1	1	1
51-60	0.66 (0.59-0.72)	0.67 (0.60-0.73)	0.65 (0.59-0.72)
>60	0.69 (0.62-0.75)	0.70 (0.64-0.77)	0.69 (0.62-0.76)
BMI: <25 (ref)	1	1	1
25-<30	1.32 (1.18-1.47)	1.33 (1.19-1.48)	1.30 (1.17-1.45)
≥30	1.65 (1.46-1.86)	1.63 (1.45-1.84)	1.65 (1.47-1.85)
Ethnicity: not white	1.29 (1.09-1.52)	1.28 (1.09-1.52)	1.27 (1.08-1.50)
Quals: College/University	0.62 (0.56-0.68)	0.61 (0.56-0.67)	0.62 (0.56-0.68)
Townsend Index: Quintile 1 (ref)	1	1	1
Quintile 2	1.00 (0.88-1.14)	1.01 (0.89-1.15)	1.02 (0.90-1.16)
Quintile 3	1.08 (0.95-1.22)	1.09 (0.96-1.24)	1.07 (0.94-1.21)
Quintile 4	1.14 (1.01-1.30)	1.08 (0.95-1.22)	1.12 (0.99-1.27)
Quintile 5	1.37 (1.21-1.55)	1.36 (1.20-1.53)	1.38 (1.22-1.56)

* End of follow-up was restricted to 30 November 2020 because there were relatively few events from 1-8 December 2020 (see Supplementary Methods).
** Quintile boundaries: Testosterone (nmol/L) Q1/2 9.0, Q2/3 10.8 , Q3/4 12.5 and Q4/5 14.8 or (ng/dL) Q1/2 259, Q2/3 311, Q3/4 360 and Q4/5 427; SHBG (nmol/L) Q1/2 25.8, Q2/3 33.1, Q3/4 40.5 and Q4/5 50.8; cFT (pmol/L) Q1/2 171, Q2/3 201, Q3/4 230 and Q4/5 268.

Table S7. Incidence Rate Ratios (IRRs) and IRR 95% confidence intervals of SARS-CoV-2 infections in UK Biobank men during follow-up (16 March 2020 – 28 February 2021).

	Hormone term modelled as quintile categories*		
**Predictor**	**Testosterone**	**SHBG**	**cFT**
Hormone: Quintile 5 (ref)	1	1	1
Quintile 4	1.01 (0.93-1.10)	1.06 (0.97-1.16)	1.06 (0.97-1.14)
Quintile 3	0.98 (0.90-1.07)	1.01 (0.92-1.10)	0.99 (0.91-1.07)
Quintile 2	0.98 (0.90-1.07)	1.06 (0.97-1.16)	1.04 (0.95-1.13)
Quintile 1	1.03 (0.94-1.12)	1.04 (0.95-1.14)	1.03 (0.94-1.13)
Age: ≤50 (ref)	1	1	1
51-60	0.62 (0.58-0.66)	0.64 (0.60-0.68)	0.63 (0.59-0.67)
>60	0.55 (0.52-0.59)	0.57 (0.53-0.61)	0.55 (0.51-0.59)
BMI: <25 (ref)	1	1	1
25-<30	1.29 (1.20-1.39)	1.29 (1.20-1.39)	1.28 (1.19-1.38)
≥30	1.64 (1.51-1.78)	1.62 (1.49-1.76)	1.65 (1.53-1.78)
Ethnicity: not white	1.69 (1.53-1.87)	1.70 (1.54-1.88)	1.70 (1.54-1.88)
Quals: College/University	0.68 (0.64-0.73)	0.68 (0.64-0.72)	0.68 (0.64-0.72)
Townsend Index: Quintile 1 (ref)	1	1	1
Quintile 2	0.99 (0.90-1.08)	0.99 (0.91-1.09)	1.00 (0.91-1.09)
Quintile 3	1.06 (0.97-1.16)	1.07 (0.98-1.16)	1.05 (0.96-1.14)
Quintile 4	1.16 (1.07-1.27)	1.12 (1.03-1.22)	1.15 (1.05-1.25)
Quintile 5	1.38 (1.27-1.50)	1.37 (1.26-1.49)	1.39 (1.27-1.51)

* Quintile boundaries: Testosterone (nmol/L) Q1/2 9.0, Q2/3 10.8, Q3/4 12.5 and Q4/5 14.8 or (ng/dL) Q1/2 259, Q2/3 311, Q3/4 360 and Q4/5 427; SHBG (nmol/L) Q1/2 25.8, Q2/3 33.1, Q3/4 40.5 and Q4/5 50.8; cFT (pmol/L) Q1/2 171, Q2/3 201, Q3/4 230 and Q4/5 268.

Table S8. Results from sensitivity analysis after excluding participants with diabetes: Hazard ratios estimating the relative risk of death from COVID-19 associated with baseline hormone concentration.[§]

Model	Q1 (lowest)	Q2	Q3	Q4	Q5 (highest)	P-value (term)
Total testosterone (nmol/L)						
Events per quintile:	76	70	73	57	67	
n per quintile:	27,750	29,652	30,225	30,471	30,658	
Model 1[#]	1.24 (0.96-1.60)	1.02 (0.78-1.39)	0.9 (0.74-1.14)	0.86 (0.78-0.99)	ref.	0.002
Model 2[##]	0.85 (0.62-1.15)	0.83 (0.62-1.15)	0.81 (0.66-1.05)	0.83 (0.74-0.95)	ref.	0.019
SHBG (nmol/L)						
Events per quintile:	51	55	68	69	100	
n per quintile:	28,436	29,684	30,081	30,263	30,292	
Model 1[#]	1.40 (1.01-1.85)	1.10 (0.82-1.45)	0.95 (0.75-1.20)	0.89 (0.80-0.99)	ref.	<0.001
Model 2[##]	1.01 (0.72-1.38)	0.88 (0.64-1.18)	0.83 (0.64-1.05)	0.84 (0.75-0.94)	ref.	0.002
cFT (pmol/L)						
Events per quintile:	103	86	61	53	40	
n per quintile:	28,275	29,516	30,044	30,292	30,629	
Model 1[#]	1.10 (0.84-1.54)	0.90 (0.69-1.29)	0.87 (0.71-1.19)	0.90 (0.82-1.20)	ref.	0.014
Model 2[##]	0.89 (0.66-1.27)	0.83 (0.63-1.19)	0.85 (0.68-1.16)	0.90 (0.82-1.19)	ref.	0.395

[§] = Hazard Ratios calculated for the medians of testosterone within each sample quintile (Q1-Q5), relative to the median for Q5.
Quintile boundaries: Testosterone (nmol/L) Q1/2 9.0, Q2/3 10.8, Q3/4 12.5 and Q4/5 14.8 or (ng/dL) Q1/2 259, Q2/3 311, Q3/4 360 and Q4/5 427; SHBG (nmol/L) Q1/2 25.8, Q2/3 33.1, Q3/4 40.5 and Q4/5 50.8; cFT (pmol/L) Q1/2 171, Q2/3 201, Q3/4 230 and Q4/5 268.
[#] = Model 1 included terms for testosterone and age and region, with time modelled as a 3-level stratification factor plus an interaction of region with time (see Methods).
[##] = Model 2 included Model 1 terms + ethnicity (white vs not white), living with partner, educational attainment, alcohol consumption, smoking status, diet (red meat: high vs low vs none), physical activity, BMI, waist circumference, cholesterol, time blood sample collected, blood type, Townsend Index quintile, hypertension, angina, atrial fibrillation, COPD, renal impairment, liver disease, thyroid disease, and use of lipid medications (a proxy for hyperlipidemia), glucocorticoids, opioids, and anticonvulsants, with the number of medications included as a proxy for overall comorbidity status. Continuous variables modelled using restricted cubic splines.

Table S9. Fully adjusted hazard ratios estimating the relative risk of death from COVID-19 associated with baseline total testosterone concentration (nmol/L), as estimated from analyses using alternative end of follow-up dates: 31 January 2021 (the main analysis); 8 December 2020; 28 February 2021.[§#]

Model	Q1 (lowest)	Q2	Q3	Q4	Q5 (highest)	P-value (term)
End of follow-up: 31 Jan 2021 (main analysis)						
Events per quintile:	112	94	86	68	78	
n per quintile:	31,992	32,169	32,081	31,933	31,789	
Hazard Ratio (95% CI):	0.84 (0.65-1.12)	0.82 (0.63-1.10)	0.80 (0.66-1.00)	0.82 (0.75-0.93)	ref.	0.008
End of follow-up: 8 Dec 2020 (up to first vaccination)						
Events per quintile:	67	63	66	52	51	
n per quintile:	31,992	32,169	32,081	31,933	31,789	
Hazard Ratio (95% CI):	0.77 (0.55-1.09)	0.87 (0.63-1.25)	0.90 (0.70-1.19)	0.90 (0.80-1.08)	ref.	0.209
End of follow-up: 28 Feb 2021 (including the tail of Wave 2)						
Events per quintile:	133	105	94	76	84	
n per quintile:	31,992	32,169	32,081	31,933	31,789	
Hazard Ratio (95% CI):	0.90 (0.71-1.18)	0.85 (0.66-1.12)	0.83 (0.69-1.01)	0.84 (0.76-0.95)	ref.	0.014

[§] = Hazard Ratios calculated for the medians of testosterone within each sample quintile (Q1-Q5), relative to the median for Q5.
Quintile boundaries: (nmol/L) Q1/2 9.0, Q2/3 10.8, Q3/4 12.5 and Q4/5 14.8 or (ng/dL) Q1/2 259, Q2/3 311, Q3/4 360 and Q4/5 427.
[#] = Presented estimates from Model 2, which included terms for testosterone and age and region, with time modelled as a stratification factor an interaction of region with time, ethnicity (white vs not white), living with partner, educational attainment, alcohol consumption, smoking status, diet (red meat: high vs low vs none), physical activity, BMI, waist circumference, cholesterol, time blood sample collected, blood type, Townsend Index quintile, diabetes, hypertension, angina, atrial fibrillation, COPD, renal impairment, liver disease, thyroid disease, and use of lipid medications (a proxy for hyperlipidemia), glucocorticoids, opioids, and anticonvulsants, with the number of medications included as a proxy for overall comorbidity status. Continuous variables modelled using restricted cubic splines (see Methods).

Table S10. Fully adjusted hazard ratios estimating the relative risk of death from COVID-19 associated with baseline SHBG concentration (nmol/L), as estimated from analyses using alternative end of follow-up dates: 31 January 2021 (the main analysis); 8 December 2020; 28 February 2021.[§#]

Model	Q1 (lowest)	Q2	Q3	Q4	Q5 (highest)	P-value (term)
End of follow-up: 31 Jan 2021 (main analysis)						
Events per quintile:	76	79	87	86	110	
n per quintile:	31,980	32,121	32,054	31,982	31,827	
Hazard Ratio (95% CI):	1.01 (0.77-1.34)	0.94 (0.72-1.24)	0.89 (0.71-1.11)	0.87 (0.79-0.97)	ref.	0.008
End of follow-up: 8 Dec 2020 (up to first vaccination)						
Events per quintile:	52	53	57	54	83	
n per quintile:	31,980	32,121	32,054	31,982	31,827	
Hazard Ratio (95% CI):	1.03 (0.73-1.44)	0.90 (0.65-1.26)	0.85 (0.67-1.11)	0.86 (0.77-0.98)	ref.	0.032
End of follow-up: 28 Feb 2021 (including the tail of Wave 2)						
Events per quintile:	87	90	93	92	130	
n per quintile:	31,980	32,121	32,054	31,982	31,827	
Hazard Ratio (95% CI):	0.93 (0.71-1.20)	0.85 (0.66-1.09)	0.83 (0.68-1.02)	0.85 (0.78-0.95)	ref.	0.005

[§] = Hazard Ratios calculated for the medians of SHBG within each sample quintile (Q1-Q5), relative to the median for Q5.
Quintile boundaries: (nmol/L) Q1/2 25.8, Q2/3 33.1, Q3/4 40.5 and Q4/5 50.8.
[#] = Presented estimates from Model 2, which included terms for SHBG and age and region, with time modelled as a stratification factor an interaction of region with time, ethnicity (white vs not white), living with partner, educational attainment, alcohol consumption, smoking status, diet (red meat: high vs low vs none), physical activity, BMI, waist circumference, cholesterol, time blood sample collected, blood type, Townsend Index quintile, diabetes, hypertension, angina, atrial fibrillation, COPD, renal impairment, liver disease, thyroid disease, and use of lipid medications (a proxy for hyperlipidemia), glucocorticoids, opioids, and anticonvulsants, with the number of medications included as a proxy for overall comorbidity status. Continuous variables modelled using restricted cubic splines (see Methods).

Table S11. Fully adjusted hazard ratios estimating the relative risk of death from COVID-19 associated with baseline calculated free testosterone concentration (cFT; pmol/L), as estimated from analyses using alternative end of follow-up dates: 31 January 2021 (the main analysis); 8 December 2020; 28 February 2021.[§#]

Model	Q1 (lowest)	Q2	Q3	Q4	Q5 (highest)	P-value (term)
End of follow-up: 31 Jan 2021 (main analysis)						
Events per quintile:	137	110	76	66	49	
n per quintile:	31,738	32,051	32,126	32,073	31,976	
Hazard Ratio (95% CI):	0.86 (0.65-1.17)	0.81 (0.61-1.10)	0.83 (0.68-1.07)	0.89 (0.82-1.06)	ref.	0.248
End of follow-up: 8 Dec 2020 (up to first vaccination)						
Events per quintile:	90	72	54	47	36	
n per quintile:	31,738	32,051	32,126	32,073	31,976	
Hazard Ratio (95% CI):	0.77 (0.55-1.10)	0.78 (0.56-1.13)	0.83 (0.66-1.11)	0.91 (0.82-1.12)	ref.	0.521
End of follow-up: 28 Feb 2021 (including the tail of Wave 2)						
Events per quintile:	159	121	90	69	53	
n per quintile:	31,738	32,051	32,126	32,073	31,976	
Hazard Ratio (95% CI):	1.01 (0.77-1.34)	0.92 (0.70-1.22)	0.90 (0.75-1.14)	0.92 (0.85-1.10)	ref.	0.196

[§] = Hazard Ratios calculated for the medians of cFT within each sample quintile (Q1-Q5), relative to the median for Q5.
Quintile boundaries: (pmol/L) Q1/2 171, Q2/3 201, Q3/4 230 and Q4/5 268.
[#] = Presented estimates from Model 2, which included terms for cFT and age and region, with time modelled as a stratification factor an interaction of region with time, ethnicity (white vs not white), living with partner, educational attainment, alcohol consumption, smoking status, diet (red meat: high vs low vs none), physical activity, BMI, waist circumference, cholesterol, time blood sample collected, blood type, Townsend Index quintile, diabetes, hypertension, angina, atrial fibrillation, COPD, renal impairment, liver disease, thyroid disease, and use of lipid medications (a proxy for hyperlipidemia), glucocorticoids, opioids, and anticonvulsants, with the number of medications included as a proxy for overall comorbidity status. Continuous variables modelled using restricted cubic splines (see Methods).

Supplemental Figures

Figure S1. Distribution of incident SARS-2-CoV events for the whole cohort. Incident events are either the first positive test or a death from COVID-19 without prior positive test, and are calculated for males and females combined, excluding participants lost to follow-up before 16 March 2020 and events after 31 Jan 2021. Squares with darker shading indicate higher numbers of incident events and zeros indicate no incident events for individuals who attended that assessment centre at baseline (2006-2010) during that month of follow-up. Assessment centres are ordered top to bottom by geographic location (north to south, west to east) for showing approximate spatiotemporal patterns of incident events in the cohort.

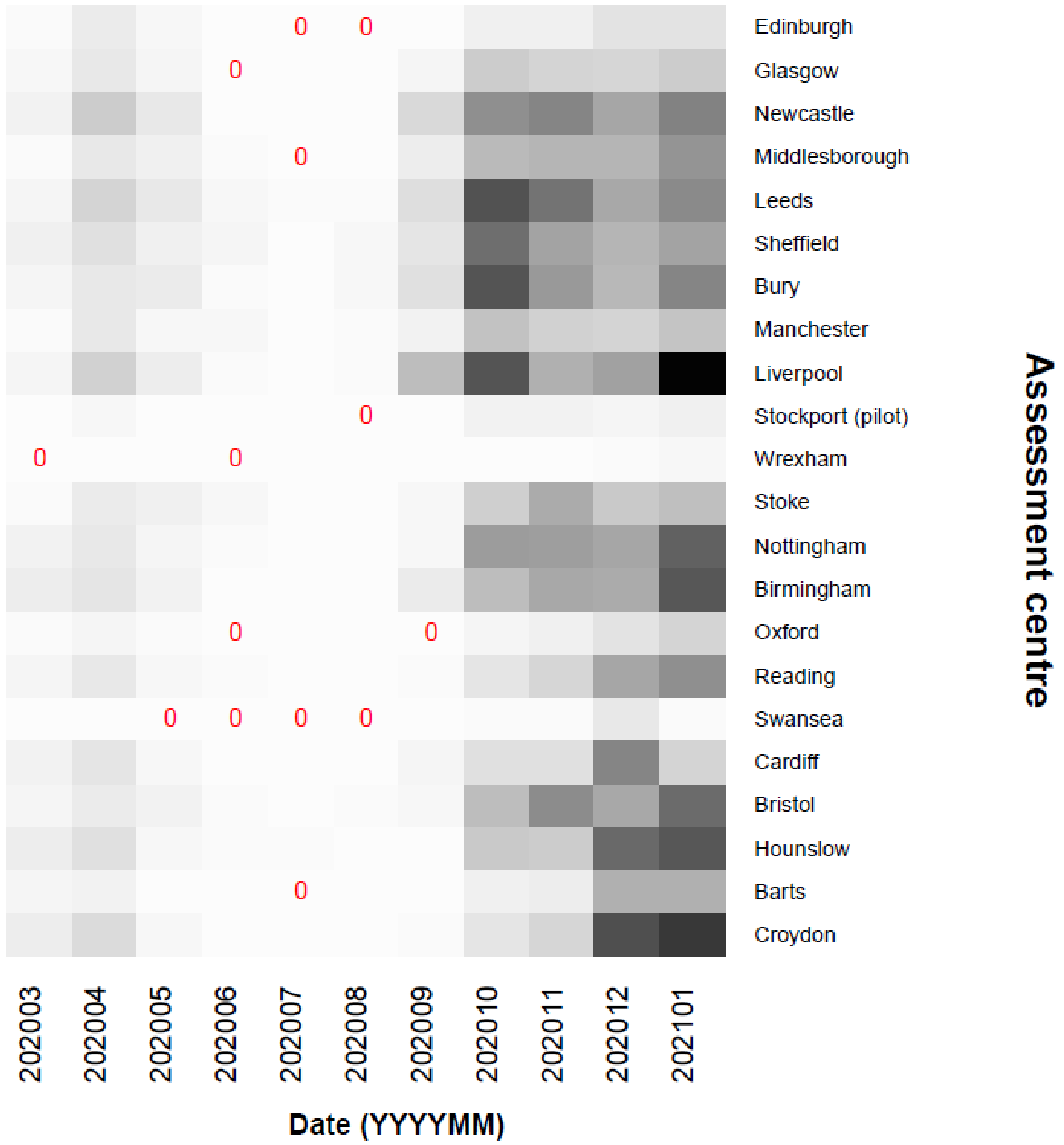

Figure S2. Derivation of the study cohort.

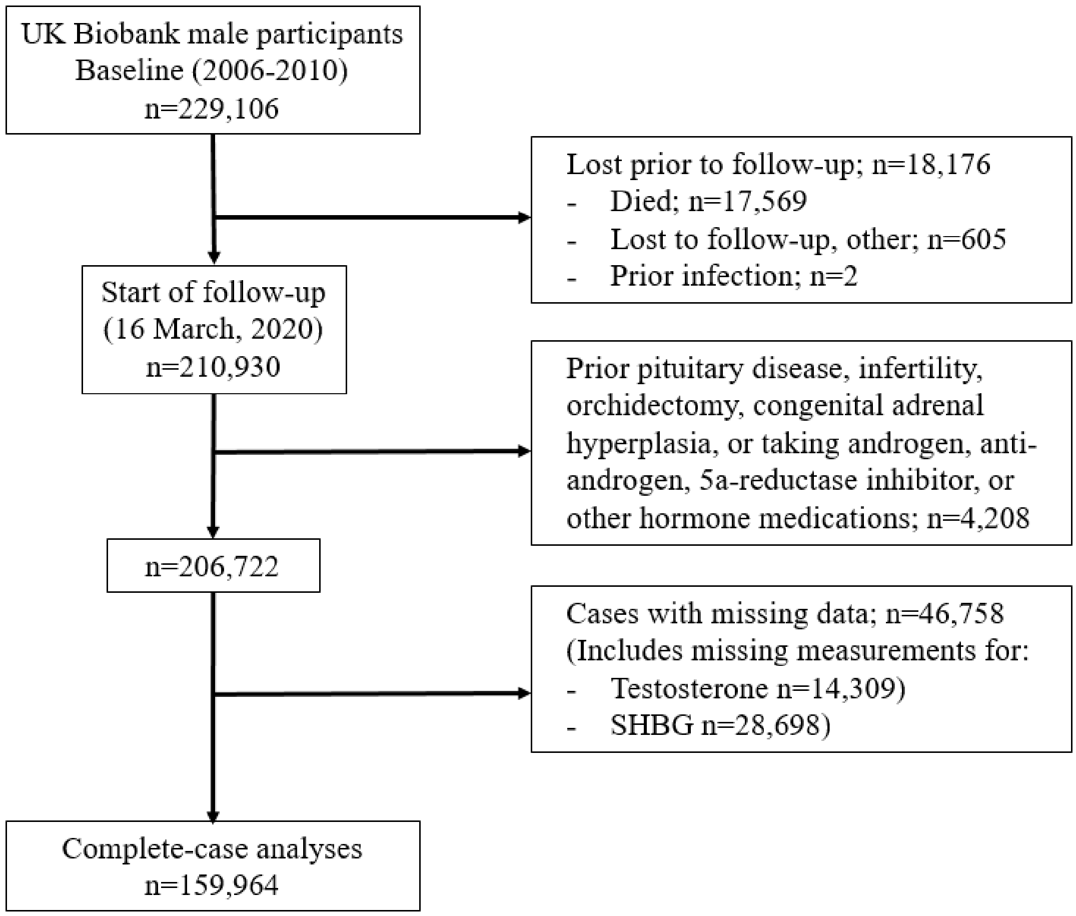

Supplement: Supplementary Material [file supplementary_material.pdf]
